# Farnesylthiosalicylic Acid Through Inhibition of Galectin‐3 Improves Neuroinflammation in Alzheimer Disease via Multiple Pathways

**DOI:** 10.1111/cns.70127

**Published:** 2024-11-26

**Authors:** Qing Qiu, Cui Li, Xiaoli Zhao, Mengting Yang, Shushu Ding, Haiying Liang, Tingting Chen

**Affiliations:** ^1^ Department of Pharmacology, School of Pharmacy Nantong University Nantong Jiangsu China; ^2^ Department of Pharmacy Longyan First Affiliated Hospital of Fujian Medical University Longyan Fujian China

**Keywords:** Alzheimer disease, farnesylthiosalicylic acid, galectin‐3, neuroinflammation, toll‐like receptors, β‐amyloid

## Abstract

**Aims:**

Many factors affect the neuroinflammatory response in patients with Alzheimer disease (AD). Galectin‐3 (Gal‐3) is closely related to microglial activation in the nervous system and can promote the aggregation of cancer cells in tumors. This study aimed to investigate the mechanism by which farnesylthiosalicylic acid (FTS) affects neuroinflammation in Aβ_1–42_ mice through Gal‐3.

**Methods:**

We used the Morris water maze, reverse transcription–polymerase chain reaction (RT–PCR), Western blotting, enzyme‐linked immunosorbent assay (ELISA), and immunofluorescence to conduct our study.

**Results:**

FTS reduced the levels of proinflammatory factors and microglial activation in Aβ_1–42_ mice. FTS inhibited total and membrane expression levels of Gal‐3 in Aβ_1–42_ mice, and the anti‐inflammatory effect of FTS was reversed by Gal‐3–adeno‐associated viral (AAV). FTS reduced the expression levels of toll‐like receptors (TLRs), effects that were reversed by Gal‐3‐AAV. Moreover, FTS ameliorated Aβ oligomerization and accumulation in Aβ_1–42_ mice, effects that were also reversed by Gal‐3‐AAV. FTS, through the inhibition of the Gal‐3–c‐Jun N‐terminal kinase (JNK) pathway, reduced PS1 expression; in addition, inhibition of Gal‐3 increased the Aβ‐degrading enzymes in Aβ_1–42_ mice. FTS‐induced improvements in cognition in Aβ_1–42_ mice were reversed by Gal‐3‐AAV.

**Conclusion:**

FTS may through inhibiting Gal‐3 reduce the expression of TLR4 and CD14 and alleviate Aβ pathology, downregulating Aβ‐stimulated TLR2, TLR4, and CD14 expression, and thus alleviate neuroinflammation in Aβ_1–42_ mice.

## Graphical abstract

FTS through inhibiting Gal‐3 reduces the expression of TLR4 and CD14, alleviates Aβ pathology, and downregulates Aβ‐stimulated TLR2, TLR4 and CD14 expression, and thus alleviates neuroinflammation in Aβ_1–42_ mice.

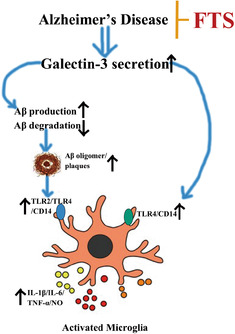

## 1 Introduction

Alzheimer disease (AD) is a common neurodegenerative disease characterized by memory loss and progressive neurocognitive dysfunction. The immune inflammatory response also plays an important role in the development and progression of AD [1, 2].

Neuroinflammation is a complex innate immune response [2, 3] produced in the central nervous system (CNS) in the face of various harmful stimuli (such as trauma or infection). Microglia are the main source of proinflammatory molecules in the brain [4]. Sustained release of proinflammatory molecules from microglia such as cytokines, chemokines, reactive nitrogen species, or reactive oxygen species can create a neurotoxic environment, driving the progression of AD [5, 6], and inflammatory molecules may further facilitate the production and aggregation of Αβ in the brain [7]. β‐Amyloid (Aβ), one of the typical pathological features of AD, is widely recognized as a key inducer of microglial activation and neuroinflammation in AD. Aβ aggregation (such as oligomeric, profibrotic, and filamentous fibril formation) and accumulation can activate microglia and promote the production of inflammatory molecules in the brain, eventually leading to AD‐associated neuroinflammation [8].

The galectin‐3 (Gal‐3) content is increased in the brain, cerebrospinal fluid, and plasma of patients with AD [9]. Gal‐3 is a member of the galectin protein family and is characterized by a single C‐terminal carbohydrate recognition domain for carbohydrate binding and an N‐terminal aggregating domain that interacts with a noncarbohydrate ligand and allows the formation of oligomers [10]. In tumors, Gal‐3 reportedly enhances cancer cell aggregation [11, 12]. Gal‐3 is expressed mainly in the cytoplasm due to the lack of a leader sequence [13] and can also be found in the nucleus and cell membrane [14]. Moreover, Gal‐3 can be released into the extracellular space upon certain stimuli, such as lipopolysaccharide (LPS) [15] and interferon γ (IFN‐γ) [16]. Gal‐3 is involved in the inflammatory response, and its expression increases in microglia upon exposure to various neuroinflammatory stimuli (e.g., in the case of ischemic injury) [17, 18, 19]. Toll‐like receptors (TLRs) are important components of the innate immune response and have been reported to participate in the Αβ‐associated inflammatory response [20, 21]. As an endogenous paracrine TLR4 ligand, Gal‐3 can bind to microglial TLR4, triggering the proinflammatory response under acute neuroinflammatory conditions.

S‐trans, trans‐farnesylthiosalicylic acid (FTS; salirasib) is a synthetic small molecule that acts as a potent Ras inhibitor. FTS dislodges all types of oncogenic Ras proteins from their membrane anchorage sites and inhibits Ras transformation both in vitro and in vivo [22, 23]. In cells, Gal‐3 serves as a scaffold for the K‐Ras protein [24]. In anaplastic thyroid carcinoma (ARO) cancer cells, FTS decreases K‐Ras, K‐Ras‐GTP, and Gal‐3 expression levels [25], and FTS disrupts the colonization of the cell membrane by Gal‐3 and Ras. After FTS treatment, Ras is mislocalized in the cytoplasm from the cell membrane. As an inhibitor of Ras activation, in the CNS, FTS has been reported to upregulate NMDA‐dependent long‐term potentiation induction and cognitive function [26], as well as neurogenesis [27], in AD mice.

This study aimed to investigate whether FTS can improve the neuroinflammatory response in AD model mice by inhibiting Gal‐3 and the possible molecular mechanisms involved to further clarify the neuroprotective effect of FTS in AD treatment.

## 2 Methods

### 2.1 Experimental Animals

This study was approved by the Experimental Animal Care and Ethical Committee of Nantong University (Approval Number: P20230221‐003). The procedures involving animals and their care were conducted in accordance with the ARRIVE Guidelines of Laboratory Animal Care. Male C57BL/6J mice aged 2 months (SLAC Laboratory Animal Co. Ltd. Shanghai, China) were housed in a constant environment (23°C ± 2°C; 55% ± 5% humidity; 12:12 h light/dark cycle) at the Animal Centre of Nantong University. The animals were given ad libitum access to food and water.

### 2.2 Drug Administration

FTS (Cayman Chemical, Ann Arbor, MI, USA) as administered by intraperitoneal (i.p.) injection at 3 mg/kg [27] starting at 4 h after Aβ_1–42_ injection for 14 days because FTS at this dose is effective and can enter the brain within 20–30 min [28]. The control mice were intraperitoneally injected with an equal volume of vehicle.

The synthetic lipoproteins FSL‐1 (100 ng/mL) and LPS (1 μg/mL) (both from InvivoGen, USA) were used as TLR2 agonists and CD14/TLR4 agonists, respectively [29].

Anisomycin (a c‐Jun N‐terminal kinase [JNK] activator) was purchased from MCE and dissolved in HCl (1 M), and the anisomycin solution was diluted with normal saline to 22 μg/μL; the pH was then adjusted to 7.4 with NaOH (5 M). For repeated intracerebroventricular (i.c.v.) injection of anisomycin (110 μg/5 μL/mouse) [30, 31, 32], a 28‐G stainless‐steel guide cannula (Plastic One, Roanoke, VA, USA) was implanted into the right cerebral ventricle and fixed on the skull [33]. Anisomycin was administered once daily for 14 consecutive days 30 min before FTS administration. The mice in the control group were injected with the same volume of vehicle (0.5% DMSO).

### 2.3 Establishment of the AD Model

The aggregation of Aβ_1–42_ in the hippocampus has been confirmed by immunostaining with an Aβ‐specific antibody [34]. Aβ_1–42_ i.c.v. injection has been shown to trigger memory impairment, synaptic disorders, tau protein hyperphosphorylation, and neurodegeneration in the mouse hippocampus [2, 35, 36, 37]. Aβ_1–42_ was dissolved in 1,1,1,3,3,3‐hexafluoro‐2‐propanol (HFIP; Sigma Aldrich), flash‐frozen in liquid nitrogen, and then lyophilized to completely remove the solvent. The lyophilized Aβ_1–42_ peptides were then dissolved in 100 mM NaOH at 6 mg/mL, aliquoted, flash‐frozen in liquid nitrogen, and stored at −80°C until use.

For i.c.v. injection of soluble Aβ_1–42_, the mice were intraperitoneally anesthetized with isoflurane and then placed in a stereotactic apparatus (Motorized Stereotaxic Stereo Drive; Neurostar). Freshly prepared Aβ_1–42_ (0.3 nmol/2 μL in 0.1 M phosphate‐buffered saline [PBS]) was injected into bilateral cerebral ventricles (0.3 mm posterior, 1.0 mm lateral, and 2.5 mm ventral to the bregma) using a stepper‐motorized microsyringe at 0.2 μL/min. In the control group, the same volume of vehicle was injected into the cerebral ventricles.

### 2.4 Morris Water Maze (MWM) Test

The MWM test was conducted for 8 consecutive days as previously reported by our group [38, 39], and the spatial cognition of the mice was assessed. The specific experimental procedures are described in Data S1.

### 2.5 Western Blotting

The animals were anesthetized and the brains were harvested, followed by separation of the hippocampus. Generation of hippocampal tissue or brain slice homogenates and protein extraction and protein imprinting experiments are described in Data S1. In this study, the primary antibodies used included mouse anti‐Aβ (6E10) (1:1000; BioLegend, Cat# 803014), anti‐Flag (Sigma, Cat# F1804), Gal‐3 (1:1000; Cat# AF1197, Bio‐techne), PS1 (1:1000; Cat# ab76083, Abcam), IDE (1:1000; Cat# ab133561, Abcam), NEP (1:1000; Cat# sc‐46,656, Santa Cruz), TTR (1:1000; Cat# sc‐377,517, Santa Cruz), anti‐phospho‐JNK (1:1000; Cat# SAB4504450, Sigma–Aldrich), and anti‐JNK (1:1000; Cat# 559304, Sigma–Aldrich). GAPDH (1:2000; Cell Signaling Technology, Cat# 5174) or β‐actin (1:2000; Cell Signaling Technology, Cat# 3700) antibodies served as internal controls. An appropriate horseradish peroxidase (HRP)‐conjugated secondary antibody was used for detection by enhanced chemiluminescence (Pierce).

### 2.6 Tissue Fixation and Immunofluorescence Staining

The methods used for tissue fixation and immunofluorescence staining of Iba‐1 and Aβ plaques are described in Data S1. The primary antibodies used included the following: anti‐Aβ (D‐11) (1:500, Cat# sc‐374,527, Santa Cruz), anti‐NeuN (1:500, Cat# ab177487, Abcam), and Iba‐1 (1:500, Cat# 019–19,741, FUJIFILM Wako). After rinsing with PBS, the sections were incubated with subtype‐specific fluorescent secondary Cy3 anti‐rabbit (Cat# 711–165‐152, Jackson ImmunoResearch) or AlexaFluor‐647 anti‐mouse (1:250, Cat# ab150107, Abcam) antibodies for 2 h at room temperature. Signal was undetectable in the control sections, which were incubated with solutions without primary antibodies.

### 2.7 Enzyme‐Linked Immunosorbent Assay (ELISA)

The levels of released interleukin‐1β (IL‐1β), IL‐6, tumor necrosis factor‐α (TNF‐α), nitric oxide (NO), and Gal‐3 were detected by ELISA, and the specific experimental procedures are described in the Data S1.

### 2.8 Virus Injections

To produce the adeno‐associated viral (AAV) vectors, the coding region of Gal‐3 was amplified from cDNA of C57BL/6J mice by PCR, and standard cloning procedures were used to subclone the enhanced green fluorescent protein (EGFP) cassettes into the backbone of AAV under a cytomegalovirus (CMV) promoter (AAV‐CMV‐MCS‐3FLAG) expression plasmid. Following DNA sequencing screening, the AAV plasmids were packaged into the AAV serotype 9 virus from GeneChem CO. Ltd. (Shanghai, China), with the titer at 1 × 10^13^ virus particles per mL.

The mice were anesthetized with isoflurane and then placed in a stereotactic apparatus. The AAV‐CMV‐Gal‐3‐EGFP (Gal‐3‐AAV) or AAV‐CMV‐EGFP control (Con‐AAV) virus were bilaterally injected into the hippocampus 2.0 mm behind the bregma and ± 1.5 mm lateral from the sagittal midline at 2 mm below the skull surface. The virus was delivered with a 10‐μL Hamilton syringe (1 μL per site) at 0.05 μL/min. The needle remained in the brain for an additional 10 min to prevent backflow of the virus suspension. The wound was closed, and the mice were allowed to recover for 3 days.

Green GFP fluorescence was detected to confirm the infection position, and the overexpression of target protein was confirmed by Western blotting of FLAG expression.

### 2.9 Reverse Transcription–Polymerase Chain Reaction (RT–PCR)

The specific experimental procedures are described in Data S1.

### 2.10 Statistical Analyses

All the statistical analyses were performed with GraphPad Prism 8. Data are presented as the mean ± standard error (S.E.M.) unless otherwise indicated. Different analyses of variance (ANOVAs) were used for comparisons of data among groups, followed by post hoc tests. A value of *p* < 0.05 was considered statistically significant.

## 3 Results

### 3.1 FTS Reduces Proinflammatory Factor Expression and Microglial Activation in Aβ_1–42_ Mice

To test the dose‐dependent effects of FTS on inflammation in Aβ_1–42_ mice, FTS (1, 3, and 10 mg/kg) was administered once daily beginning at 4 h after Aβ_1–42_ injection for 14 consecutive days (Figure S1). Based on the results of this pilot experiment, we used 3 mg/kg FTS for subsequent experiments.

**FIGURE 1:**
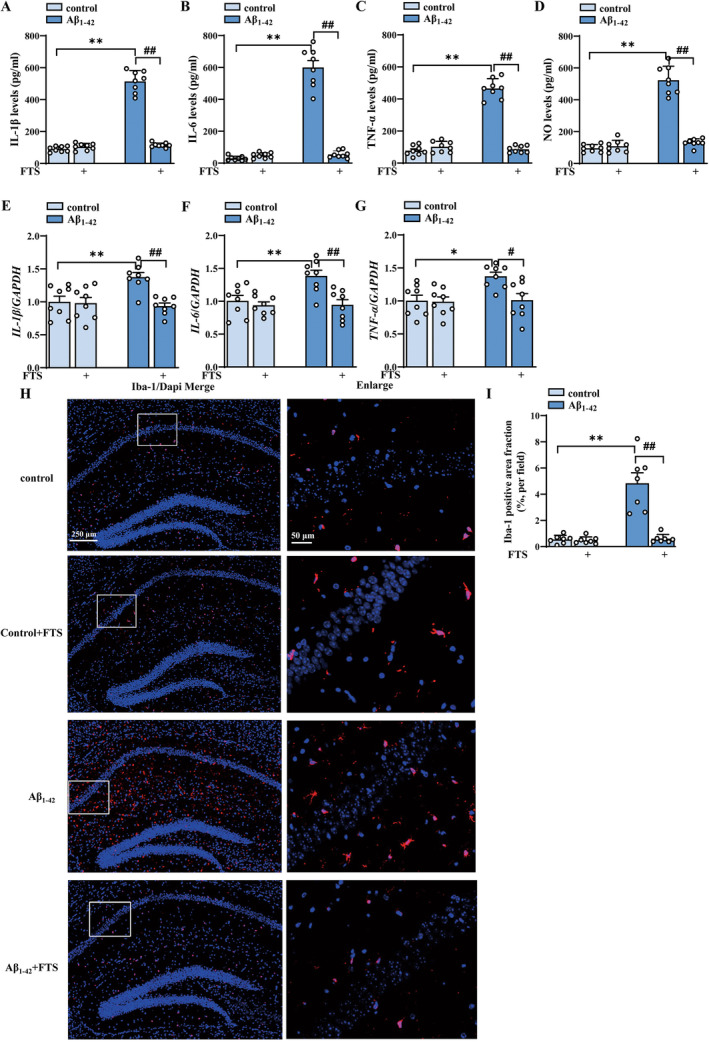
FTS reduces the expression of proinflammatory factors and microglial activation in Aβ_1–42_ mice. (A–D) Levels of the inflammatory molecules IL‐1β, IL‐6, TNF‐γ, and NO in the control mice and Aβ_1–42_ mice treated with FTS. (E–G) mRNA expression levels of the inflammatory factors IL‐1β, IL‐6, and TNF‐γ in control mice and Aβ_1–42_ mice treated with FTS. (H) Representative images of hippocampal microglia (Iba‐1; red, right) and nuclei (DAPI; blue, right), with representative images in the square in the middle (left). (I) The bar graph shows the Iba‐1‐positive area fractions (%, per field) in the control mice and Aβ_1–42_ mice (right). (A‐I) *p < 0.05 and **p < 0.01 vs. control mice, #*p* < 0.05 and ##*p* < 0.01 vs. Aβ_1–42_ mice.

As shown in Figure 1A–D, the contents of proinflammatory factors in the hippocampus were highly increased in Aβ_1–42_ mice, as detected by ELISA (IL‐1β: F (1, 28) = 275.3, *p* < 0.0001; IL‐6: F (1, 28) = 167.6, *p* < 0.0001; TNF‐α: F (1, 28) = 175.3, *p* < 0.0001; NO: F (1, 28) = 156.2, *p* < 0.0001; Aβ_1–42_ vs. control: all *p* < 0.0001, *n*  = 8 mice per group, two‐way ANOVA, followed by Sidak's test), effects that were significantly reversed by FTS treatment (IL‐1β: F (1, 28) = 214.8, *p* < 0.0001; IL‐6: F (1, 28) = 140.2, *p* < 0.0001; TNF‐α: F (1, 28) = 158.8, *p* < 0.0001; NO: F (1, 28) = 111.0, *p* < 0.0001; Aβ_1–42_ vs. Aβ_1–42_ + FTS: all *p* < 0.0001). FTS had no effect on the contents of proinflammatory factors in the control mice (*IL‐1β*: *p* = 0.9703, *IL‐6*: *p* = 0.9933, *TNF‐α*: *p* = 0.7928, *NO*: *p* = 0.997; Figure 1A–D). The mRNA expression levels of *IL‐1β*, *IL‐6*, and *TNF‐α* also increased in Aβ_1–42_ mice (*IL‐1β*: F (1, 28) = 5.095, *p* = 0.032; Aβ_1–42_ vs. control: *p* = 0.007; *IL‐6*: F (1, 28) = 6.372, *p* = 0.0175; Aβ_1–42_ vs. control: *p* = 0.0089; TNF‐α: F (1, 28) = 6.062, *p* = 0.0202; Aβ_1–42_ vs. control: *p* = 0.0167, *n* = 8 mice per group; two‐way ANOVA, followed by Sidak's test), effects that were reduced by FTS treatment (*IL‐1β*: F (1, 28) = 9.830, *p* = 0.004, Aβ_1–42_ vs. Aβ_1–42_+ FTS: *p* = 0.0013; *IL‐6*: F (1, 28) = 11.11, *p* = 0.0024, Aβ_1–42_ vs. Aβ_1–42_ + FTS: *p* = 0.0020; TNF‐α: F (1, 28) = 5.594, *p* = 0.0252, Aβ_1–42_ vs. Aβ_1–42_ + FTS: *p* = 0.0198; Figure 1E–G). Gliosis is one of the characteristic pathological events in AD [40]. In this study, the activation of microglia by Iba‐1 (a biomarker of microglia, Figure 1H) was examined. Compared with those in control mice, the Iba‐1‐positive areas in Aβ_1–42_ mice were significantly greater (F (1, 28) = 16.67, *p* = 0.0003; Aβ_1–42_ vs. control: *p* < 0.0001; *n* = 8 mice per group; two‐way ANOVA followed by Sidak's test), effects that were reduced by FTS treatment (F (1, 28) = 17.24, *p* = 0.0003, Aβ_1–42_ vs. Aβ_1–42_ + FTS: *p* < 0.0001; Figure 1I). These results indicate that FTS inhibits the overactivation of microglia in Aβ_1–42_ mice.

### 3.2 Gal‐3 Expression Increases in Aβ_1–42_ Mice but is Inhibited by FTS

**FIGURE 2:**
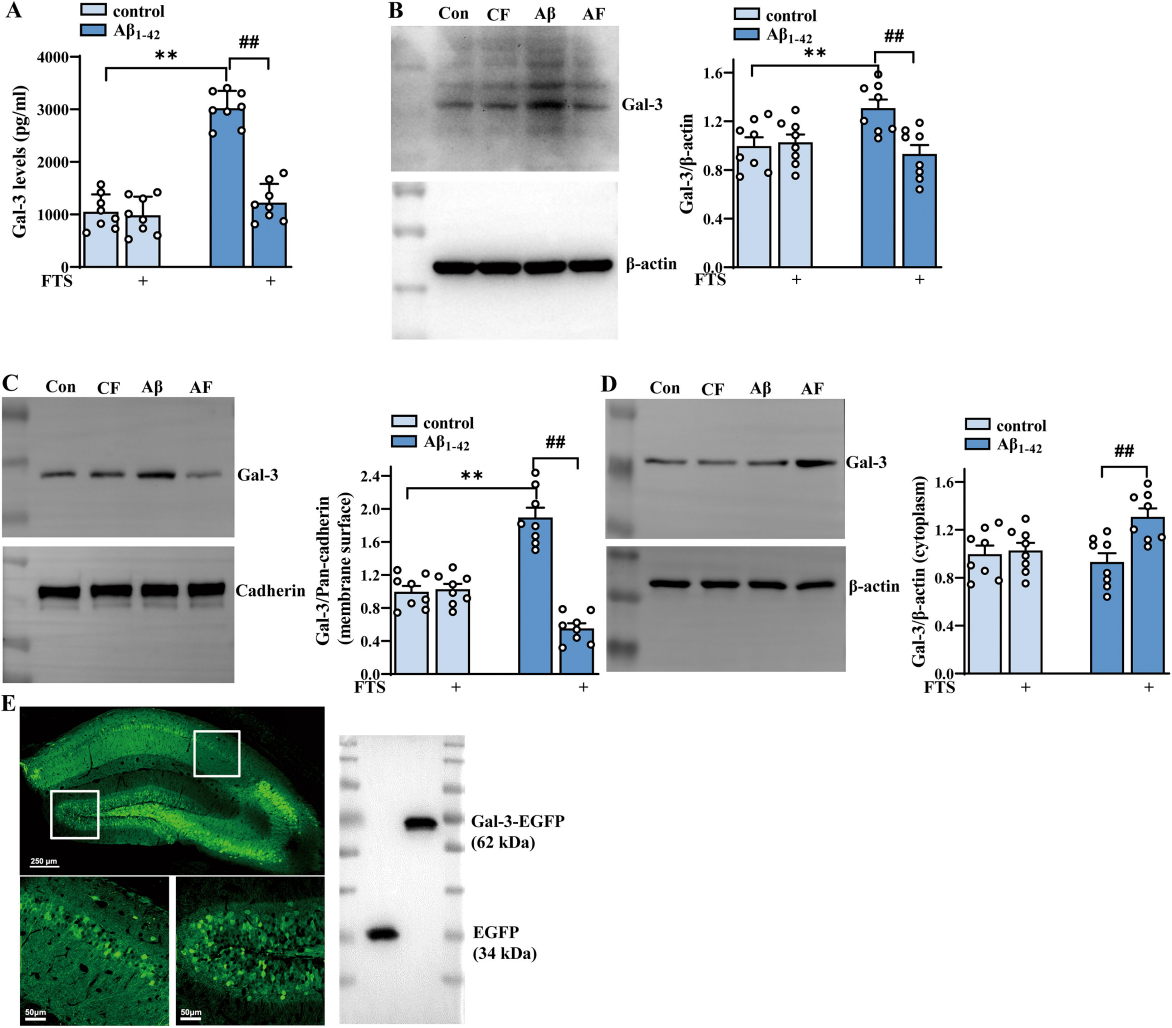
Gal‐3 expression is increased in Aβ_1–42_ mice but is inhibited by FTS. (A) Hippocampal Gal‐3 contents in control and Aβ_1–42_ mice treated with FTS. (B) Left: Representative images of total Gal‐3 and β‐actin (internal reference); right: Bar graphs showing the total expression of Gal‐3 in the hippocampus of control mice and Aβ_1–42_ mice treated with FTS. (C) Left: Representative image of membrane Gal‐3 and pan‐cadherin (internal reference); right: Bar graphs showing the membrane expression levels of Gal‐3 in the hippocampus of control mice and Aβ_1–42_ mice treated with FTS. (D) Left: Representative image of the cytoplasmic expression levels of Gal‐3 and β‐actin (internal reference); right: Bar graphs showing the cytoplasmic expression levels of Gal‐3 in the hippocampus of control mice and Aβ_1–42_ mice treated with FTS. (A‐D) **p < 0.01 vs. control mice, ##*p* < 0.01 vs. Aβ_1–42_. (E) Hippocampal tissue infected with Gal‐3‐AAV (left) and showed considerable Flag expression (EGFP + Flag and Gal‐3 + EGFP + Flag, right).

To explore whether the increases in inflammatory factor levels and microglial activation in Aβ_1–42_ mice were related to the increased release of Gal‐3, the content of endogenous Gal‐3 was detected by ELISA. The results revealed that the hippocampal Gal‐3 content significantly increased in Aβ_1–42_ mice (F (1, 28) = 83.55, *p* < 0.0001; Aβ_1–42_ vs. control: *p* < 0.0001; *n* = 8 mice per group; two‐way ANOVA, followed by Sidak's test), which was reversed by FTS treatment (F (1, 28) = 59.23, *p* < 0.0001, Aβ_1–42_ vs. Aβ_1–42_ + FTS: *p* < 0.0001; Figure 2A). The results also revealed that both total and membrane levels of Gal‐3 increased in Aβ_1–42_ mice (total: F (1, 28) = 5.290, *p* = 0.0291, Aβ_1–42_ vs. control: *p* = 0.0013, Figure 2B; membrane: F (1, 28) = 5.545, *p* = 0.0258, Aβ_1–42_ vs. control: *p* < 0.0001, Figure 2C; *n* = 8 mice per group, two‐way ANOVA, followed by Sidak's test), effects that were reduced by FTS treatment (total: F (1, 28) = 10.62, *p* = 0.0029, Aβ_1–42_ vs. Aβ_1–42_ + FTS: *p* = 0.0002, Figure 2B; membrane: F (1, 28) = 70.14, *p* < 0.0001, Aβ_1–42_ vs. Aβ_1–42_ + FTS: *p* < 0.0001; Figure 2C). Notably, the membrane level of Gal‐3 in the FTS‐treated Aβ_1–42_ mice was markedly lower than that in the control mice (*p =* 0.0011). However, the cytoplasmic level of Gal‐3 remained unchanged in Aβ_1–42_ mice (F (1, 28) =2.370, *p* = 0.1349, Aβ_1–42_ vs. control: *p* = 0.9103, *n* = 8 mice per group; two‐way ANOVA followed by Sidak's test), but it increased significantly after FTS treatment (F (1, 28) = 8.450, *p* = 0.0071, Aβ_1–42_ vs. Aβ_1–42_ + FTS: *p* = 0.0037; Figure 2D). To investigate whether the effect of FTS on inflammatory molecules in Aβ_1–42_ mice was related to Gal‐3, Gal‐3‐AAV was constructed to induce Gal‐3 overexpression in mice. As shown in Figure 2E, Gal‐3‐AAV effectively infected the hippocampus and produced considerable FLAG expression (Figure 2E, right). Overall, we speculated that for Aβ_1–42_ mice, FTS might reduce membrane expression but enhance the cytoplasmic expression of Gal‐3, leading to its degradation and subsequent downregulation of total expression.

### 3.3 The Anti‐Inflammatory Effect of FTS is Dependent on Gal‐3 and is Related to TLRs

**FIGURE 3:**
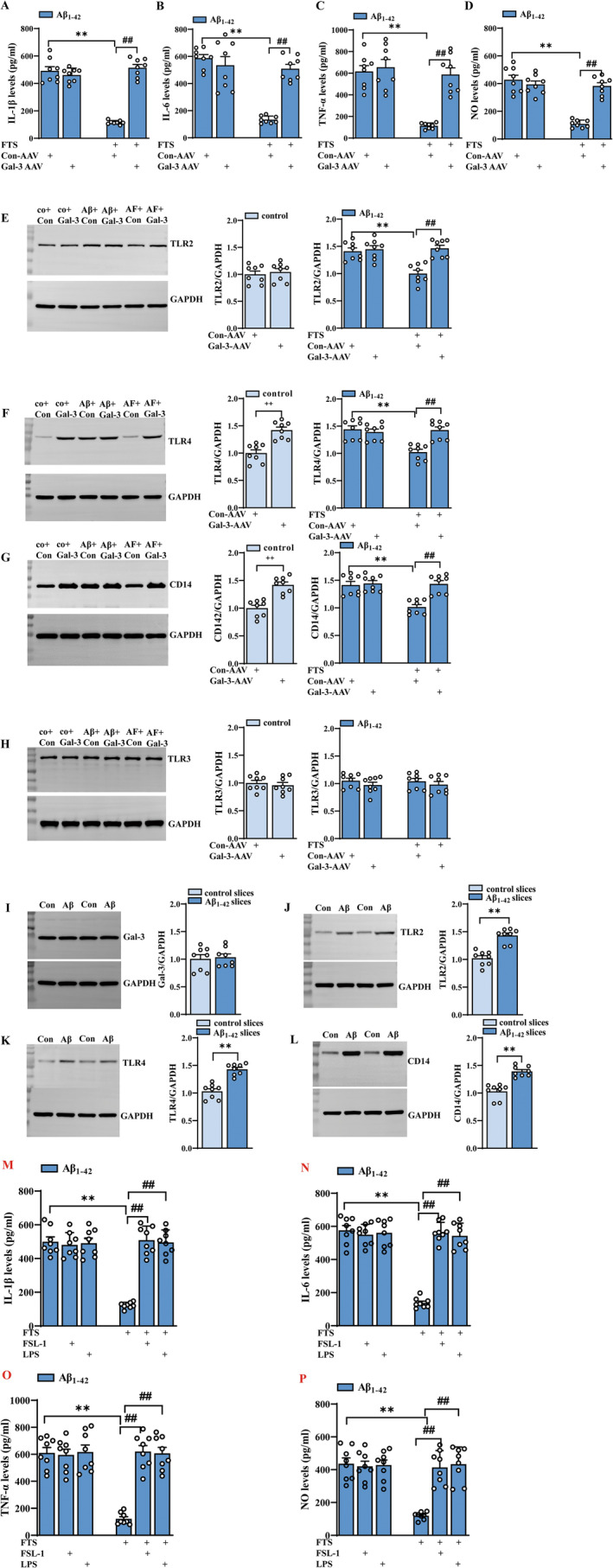
The anti‐inflammatory effect of FTS is dependent on Gal‐3 and is related to TLRs. (A–D) Levels of the inflammatory molecules IL‐1β, IL‐6, TNF‐γ, and NO in Aβ_1–42_ mice with or without FTS and Gal‐3‐AAV treatment. **p < 0.01 vs. Aβ_1–42_ mice, ##*p* < 0.01 vs. Aβ_1–42_ mice treated with FTS. (E–H) Expression levels of the TLR2, LTR4, CD14, and TLR3 proteins in control mice treated with or without Gal‐3‐AAV and in Aβ_1–42_ mice treated with or without FTS and Gal‐3‐AAV. ++*p* < 0.01 vs. control mice; **p < 0.01 vs. Aβ_1–42_ mice; ##*p* < 0.01 vs. Aβ_1–42_ mice treated with FTS. (I–L) Expression levels of Gal‐3‐AAV, TLR2, LTR4, and CD14 proteins in slices treated with or without Aβ_1–42_ for 4 h. **p < 0.01 vs. control slices. (M–P) Levels of the inflammatory molecules IL‐1β, IL‐6, TNF‐γ, and NO in Aβ_1–42_ mice with or without FTS, FSL‐1, and LPS treatment. **p < 0.01 vs. Aβ_1–42_ mice, ##*p* < 0.01 vs. Aβ_1–42_ mice treated with FTS.

As shown by the ELISA results in Figure 3, the reduced contents of IL‐1β, IL‐6, TNF‐α, and NO induced by FTS treatment in Aβ_1–42_ mice were reversed in the presence of Gal‐3 overexpression (IL‐1β: F (1, 28) = 72.50, *p* < 0.0001; IL‐6: F (1, 28) = 18.02, *p* = 0.0002; TNF‐α: F (1, 28) = 21.87, *p* < 0.0001; NO: F (1, 28) = 24.95, *p* < 0.0001; Aβ_1–42_ + FTS vs. Aβ_1–42_ + FTS + Gal‐3‐AAV: all *p* < 0.0001; *n* = 8 mice per group, two‐way ANOVA, followed by Sidak's test; Figure 3A–D), which indicates the key role of Gal‐3 in the FTS‐induced inhibition of inflammation in Aβ_1–42_ mice.

In mammals, there are at least 10 TLRs, and some employ additional coreceptors that assist in pathogen recognition, such as CD14 for TLR4 [41]. In human AD brains, both TLR2‐ and CD14‐positive microglia are associated with Aβ plaques [42]. Higher TLR4 mRNA expression was reported in the TgCRND8 mouse model of AD [43]. TLR3 is expressed mainly in the brain and can participate in the regulation of the immune response, nerve regeneration, and neuronal plasticity [44]. We found that FTS treatment could significantly reduce the expression levels of TLR2, TLR4, and CD14 in Aβ_1–42_ mice (TLR2: F (1, 28) = 10.75, *p* = 0.0028, Aβ_1–42_ vs. Aβ_1–42_ + FTS: *p* = 0.0002, Figure 3E; TLR4: F (1, 28) = 11.34, *p* = 0.0022, Aβ_1–42_ vs. Aβ_1–42_ + FTS: *p* < 0.0001, Figure 3F; CD14: F (1, 28) = 13.37, *p* = 0.0010, Aβ_1–42_ vs. control: *p* = 0.0001, *n* = 8 mice per group, two‐way ANOVA, followed by Sidak's test; Figure 3G), and these effects of FTS were reversed by Gal‐3 overexpression (TLR2: F (1, 28) = 17.25, *p* = 0.0003, Aβ_1–42_ + FTS vs. Aβ_1–42_ + FTS + Gal‐3‐AAV: *p* < 0.0001; TLR4: F (1, 28) = 9.636 *p* = 0.0043, Aβ_1–42_ + FTS vs. Aβ_1–42_ + FTS + Gal‐3‐AAV: *p* = 0.0002; CD14: F (1, 28) = 16.12, *p* = 0.0004, Aβ_1–42_ + FTS vs. Aβ_1–42_ + FTS + Gal‐3‐AAV: *p* < 0.0001). In contrast, the expression levels of TLR4 and CD14 (TLR4: *t* = 5.164, df = 14, *p* = 0.0001; Figure 3F; CD14: *t* = 5.657, df = 14, *p* < 0.0001; unpaired Student's *t* test; Figure 3G), but not that of TLR2 (TLR2: *t* = 0.5264, df = 14, *p* = 0.6068; unpaired Student's *t* test; Figure 3E), were upregulated in Gal‐3‐AAV‐infected control mice. TLR3 expression was not influenced by FTS in Aβ_1–42_ mice (F (1, 28) = 0.00058, *p* = 0.9808, *n* = 8 mice per group; two‐way ANOVA followed by Sidak's test; Figure 3H) or by Gal‐3 overexpression in control mice (TLR2: *t* = 0.5731, df = 14, *p* = 0.5757; unpaired Student's *t* test; Figure 3H). These results indicate that FTS could reduce the increased levels of TLR2, TLR4, and CD14 through the inhibition of Gal‐3 in Aβ_1–42_ mice. On the one hand, Gal‐3 could regulate TLR4 and CD14 expression; on the other hand, considering the effect of Aβ on TLRs, we speculated that Gal‐3 could regulate TLRs by affecting Aβ pathologies. Therefore, we examined the levels of TLRs in Aβ‐incubated hippocampal slices. We found that after Aβ incubation for 4 h, the expression of Gal‐3 did not change (*t* = 0.3003, df = 14, *p* = 0.7684, unpaired Student's *t* test; Figure 3I); in contrast, the levels of TLR2, TLR4, and CD14 did increase (TLR2: *t* = 6.372, df = 14, *p* < 0.0001; TLR4: *t* = 6.747, df = 14, *p* < 0.0001; CD14: *t* = 6.205, df = 14, *p* < 0.0001, unpaired Student's *t* test; Figure 3J–L). Therefore, in Aβ_1–42_ mice, FTS could reduce the expression levels of TLR4 and CD14 through the inhibition of Gal‐3; meanwhile, FTS could downregulate TLR2, TLR4, and CD14 through affecting Aβ pathologies, effects that were also dependent on Gal‐3.

Moreover, the reduced contents of IL‐1β, IL‐6, TNF‐α, and NO in Aβ_1–42_ mice were reversed by the application of the TLR2 agonist FSL‐1 and the CD14/TLR4 agonist LPS (IL‐1β: F (2, 42) = 34.60, *p* < 0.0001, Aβ_1–42_ + FTS vs. Aβ_1–42_ + FTS + FSL‐1: *p* < 0.0001; Aβ_1–42_ + FTS vs. Aβ_1–42_ + FTS + LPS: *p* < 0.0001; IL‐6: F (2, 42) = 46.29, *p* < 0.0001, Aβ_1–42_ + FTS vs. Aβ_1–42_ + FTS + FSL‐1: *p* < 0.0001; Aβ_1–42_ + FTS vs. Aβ_1–42_ + FTS + LPS: *p* < 0.0001; TNF‐α: F (2, 42) = 23.03, *p* < 0.0001, Aβ_1–42_ + FTS vs. Aβ_1–42_ + FTS + FSL‐1: *p* < 0.0001; Aβ_1–42_ + FTS vs. Aβ_1–42_ + FTS + LPS: *p* < 0.0001; NO: F (2, 42) = 14.46, *p* < 0.0001, Aβ_1–42_ + FTS vs. Aβ_1–42_ + FTS + FSL‐1: *p* < 0.0001; Aβ_1–42_ + FTS vs. Aβ_1–42_ + FTS + LPS: *p* < 0.0001; *n* = 8 mice per group, two‐way ANOVA, followed by Tukey's test; Figure 3M–P), which indicated that FTS regulated inflammatory markers in Aβ_1–42_ mice by affecting TLR2 and CD14/TLR4.

### 3.4 Aβ Injection‐Induced Aβ Oligomerization and Accumulation are Ameliorated by FTS, Which is Reversed by Gal‐3 Overexpression

**FIGURE 4:**
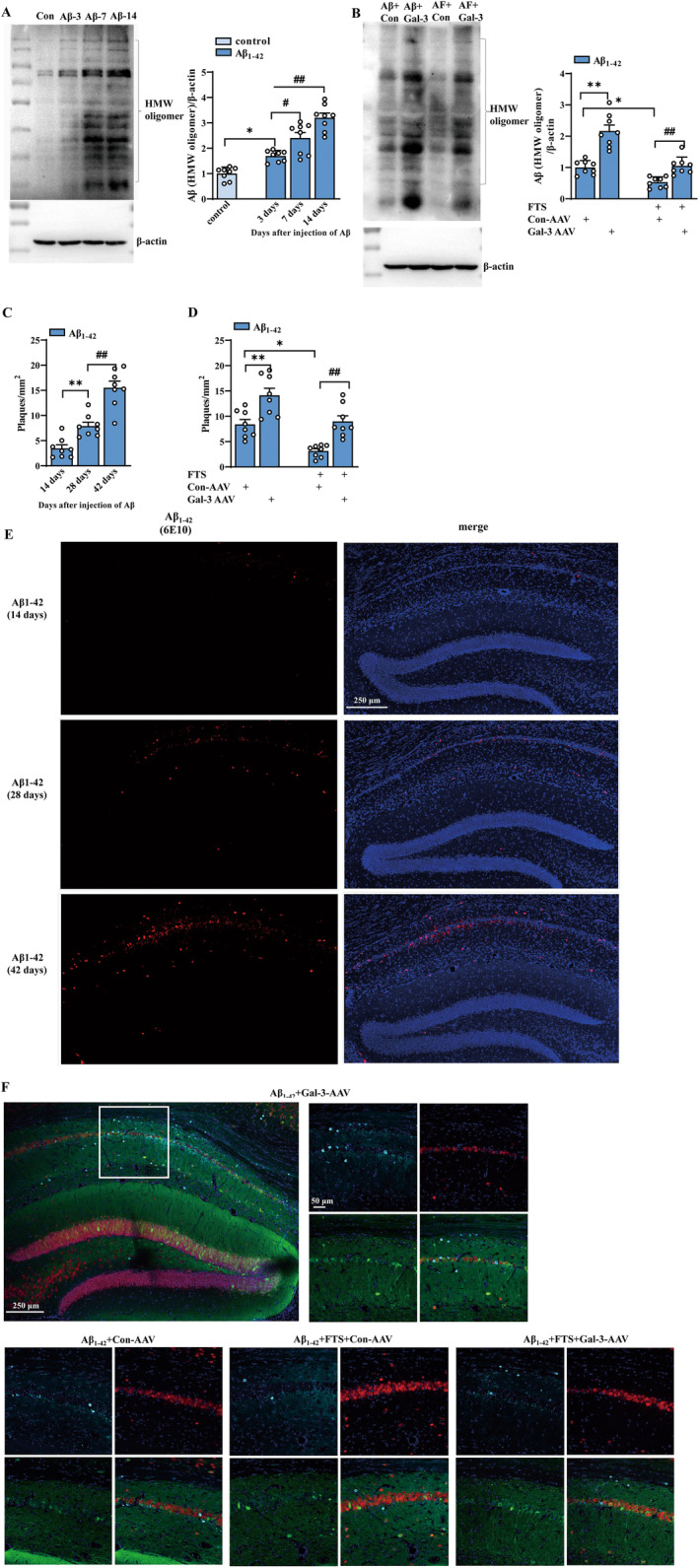
Aβ injection‐induced Aβ oligomerization and accumulation are ameliorated by FTS, which is reversed by Gal‐3 overexpression. (A) Amounts of Aβ oligomerization in mice injected with vehicle (i.c.v.) (control mice) for 14 days and in mice treated with Aβ_1–42_ (i.c.v.) (Aβ_1–42_ mice) at 3, 7, and 14 days postinjection. *p < 0.05 vs. control mice, #*p* < 0.05 and ##*p* < 0.01 vs. Aβ_1–42_ mice (3 days); (B) Amounts of Aβ oligomerization in the Aβ_1–42_ mice with or without FTS and Gal‐3‐AAV treatment. *p < 0.05 and **p < 0.01 vs. Aβ_1–42_ mice, ##*p* < 0.01 vs. Aβ_1–42_ mice treated with FTS. (C) Bar graph showing Aβ plaques in mice treated with Aβ_1–42_ at 14, 28, and 42 days postinjection shown in (E), red signal: Aβ, blue signal: DAPI, **p < 0.01 vs. Aβ_1–42_ mice (14 days), ##*p* < 0.01 vs. Aβ_1–42_ mice (28 days). (D) Bar graph showing Aβ plaques in Aβ_1–42_ mice treated with or without FTS and Gal‐3‐AAV shown in (F). *p < 0.05 and **p < 0.01 vs. Aβ_1–42_ mice, ##*p* < 0.01 vs. Aβ_1–42_ mice treated with FTS. (E) Representative images of hippocampal Aβ plaques (red) and DAPI (blue) at 14, 28, and 42 days postinjection. (F) Representative images of hippocampal Aβ plaques (blue), NeuN (red), and Gal‐3‐AAV (green) in Aβ_1–42_ mice with or without FTS and Gal‐3‐AAV treatment.

The inhibition of Aβ aggregation has been proposed as a therapeutic strategy to alleviate the progression of AD pathology [45]. Aβ monomers form soluble oligomers with different molecular weights, and Aβ oligomers (AβOs) further aggregate to generate insoluble Aβ fibrils and amyloid plaques [46, 47]. The results revealed that the amount of high‐molecular‐weight (HMW) AβOs (> 23 kDa) increased in Aβ_1–42_ mice in a time‐dependent manner at 3, 7, and 14 days postinjection (F (3, 28) = 37.78, *p* < 0.0001; *n* = 8 mice per group; one‐way ANOVA followed by Tukey's test; Figure 4A). Aβ oligomerization was greater at 3 days in Aβ_1–42_ mice than in control mice treated with NS (*p* = 0.0174), and the number of oligomers further increased at 7 (*p* = 0.0150) and 14 days (*p* < 0.0001) compared with that at 3 days. Compared with Aβ_1–42_ mice, Aβ_1–42_ mice treated with Gal‐3‐AAV presented significantly more oligomers, and FTS treatment reduced the number of oligomers in Aβ_1–42_ mice (F (1, 28) = 47.17, *p* < 0.0001, Aβ_1–42_ vs. Aβ_1–42_ + FTS: *p* = 0.0342, *n* = 8 mice per group, two‐way ANOVA, followed by Sidak's test). Interestingly, Gal‐3‐AAV overexpression increased the number of oligomers in both Aβ_1–42_ mice and Aβ_1–42_ + FTS mice (F (1, 28) = 53.42, *p* < 0.0001; Aβ_1–42_ vs. Aβ_1–42_ + Gal‐3‐AAV: *p* < 0.0001; Aβ_1–42_ + FTS vs. Aβ_1–42_ + FTS + Gal‐3‐AAV: *p* < 0.0001; Figure 4B). Aβ_1–42_ accumulation was subsequently assessed by immunofluorescence staining. As shown in Figure 4C,E, Aβ plaques began to appear at 28 days and further increased at 42 days (F (2, 21) = 39.85, *p* < 0.0001; 14 days vs. 28 days: *p* = 0.0104; 28 days vs. 42 days: *p* < 0.0001; *n* = 8 mice per group; one‐way ANOVA, followed by Tukey's test; Figure 4C). Additionally, FTS treatment decreased the number of Aβ plaques in Aβ_1–42_ mice (F (1, 28) = 47.17, *p* < 0.0001, *p* = 0.0342; Figure 4F), and the injection of Gal‐3‐AAV increased the number of plaques in both Aβ_1–42_ mice and Aβ_1–42_ + FTS mice (F (1, 28) = 53.42, *p* < 0.0001; Aβ_1–42_ vs. Aβ_1–42_ + Gal‐3‐AAV: *p* < 0.0001; Aβ_1–42_ + FTS vs. Aβ_1–42_ + FTS + Gal‐3‐AAV: *p* < 0.0001; *n* = 8 mice per group; two‐way ANOVA, followed by Sidak's test; Figure 4D). These results indicate that Gal‐3 overexpression increases Aβ oligomerization and accumulation in Aβ_1–42_ mice and Aβ_1–42_ + FTS mice.

### 3.5 FTS Alleviates Aβ Pathology Through Reducing Aβ Production and Promoting Aβ Degradation

**FIGURE 5:**
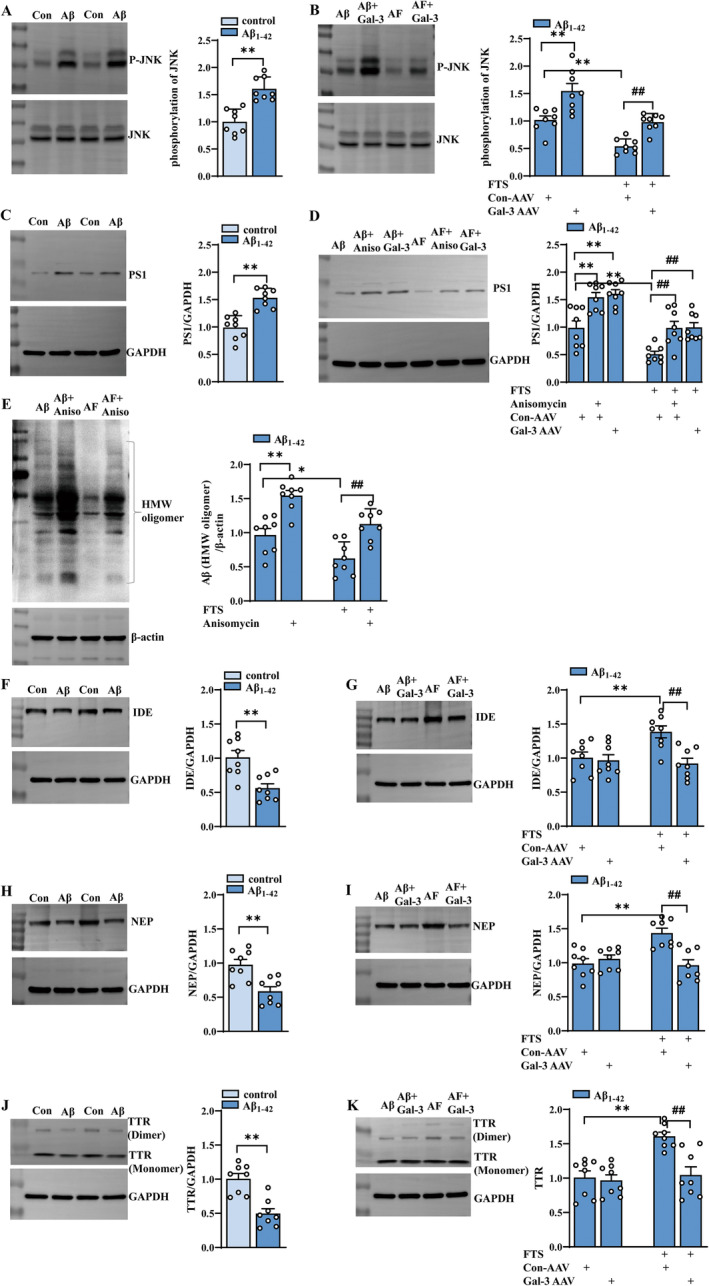
FTS alleviates Aβ pathology by reducing Aβ production and promoting Aβ degradation. (A) Expression of phospho‐JNK in the hippocampus of control mice and Aβ_1–42_‐treated mice, **p < 0.01 vs. control mice. (B) Expression of phospho‐JNK in the hippocampus of Aβ_1–42_ mice with or without FTS and Gal‐3‐AAV treatment; **p < 0.01 vs. Aβ_1–42_ mice; ##*p* < 0.01 vs. Aβ_1–42_ mice treated with FTS. (C) Expression of PS1 in the hippocampus of control mice and Aβ_1–42_‐treated mice, **p < 0.01 vs. control mice. (D) Expression of PS1 in the hippocampus of Aβ_1–42_ mice with or without FTS, anisomycin, or Gal‐3‐AAV treatment; **p < 0.01 vs. Aβ_1–42_ mice; ^##^ *p* < 0.01 vs. Aβ_1–42_ mice treated with FTS. (E) Amounts of Aβ oligomerization in Aβ_1–42_ mice with or without FTS and anisomycin treatment. *p < 0.05 and **p < 0.01 vs. Aβ_1–42_ mice, ^##^ *p* < 0.01 vs. Aβ_1–42_ mice treated with FTS. (F) Expression of IDE in the hippocampus of control mice and Aβ_1–42_‐treated mice, **p < 0.01 vs. control mice. (G) Expression of IDE in the hippocampus of Aβ_1–42_ mice with or without FTS and Gal‐3‐AAV treatment, **p < 0.01 vs. Aβ_1–42_ mice, ##*p* < 0.01 vs. Aβ_1–42_ mice treated with FTS. (H) Expression of NEP in the hippocampus of control mice and Aβ_1–42_ mice treated with vehicle; **p < 0.01 vs. control mice. (I) Expression of NEP in the hippocampus of Aβ_1–42_ mice with or without FTS and Gal‐3‐AAV treatment; **p < 0.01 vs. Aβ_1–42_ mice; ##*p* < 0.01 vs. Aβ_1–42_ mice treated with FTS. (J) Expression of TTR in the hippocampus of control mice and Aβ_1–42_ mice treated with vehicle; **p < 0.01 vs. control mice. (K) Expression of TTR in the hippocampus of Aβ_1–42_ mice with or without FTS and Gal‐3‐AAV treatment; **p < 0.01 vs. Aβ_1–42_ mice; ^##^ *p* < 0.01 vs. Aβ_1–42_ mice treated with FTS.

It has been reported that hyperactivated Ras can increase the level of phosphorylated JNK in imaginal discs [48]. PS1‐ or PS2‐containing γ‐secretase has been implicated in the development of AD because of its role in the cleavage of APP and the production of Aβ [49, 50]. A JNK‐specific inhibitor can repress PS1 expression and γ‐secretase activity in SK‐N‐SH cells in vitro and in the mouse brain in vivo [51]. Thus, we further explored whether FTS reduces PS1 through downregulating JNK phosphorylation, ultimately reducing Aβ production. The results revealed that the phosphorylation of JNK was markedly increased in Aβ_1–42_ mice (*t* = 5.388, df = 14, *p* < 0.0001; unpaired Student's *t* test; Figure 5A), an effect that was significantly reduced by FTS treatment (F (1, 28) = 38.02, *p* < 0.0001; Aβ_1–42_ vs. Aβ_1–42_ + FTS: *p* = 0.0009; *n* = 8 mice per group; two‐way ANOVA followed by Sidak's test), and Gal‐3 overexpression increased the phosphorylation of JNK in both Aβ_1–42_ mice and Aβ_1–42_ + FTS mice (F (1, 28) = 32.41, *p* < 0.0001; Aβ_1–42_ vs. Aβ_1–42_ + Gal‐3‐AAV: *p* < 0.0001; Aβ_1–42_ + FTS vs. Aβ_1–42_ + FTS + Gal‐3‐AAV: *p* = 0.0021; Figure 5B). The expression of PS1 was significantly upregulated in Aβ_1–42_ mice (*t* = 5.522, df = 14, *p* < 0.0001, unpaired Student's *t* test; Figure 5C), which decreased after FTS treatment (F (1, 42) = 50.62, *p* < 0.0001, Aβ_1–42_ vs. Aβ_1–42_ + FTS: *p* = 0.0027, *n* = 8 mice per group, two‐way ANOVA, followed by Sidak's or Tukey's test; Figure 5D). Gal‐3 overexpression and anisomycin enhanced PS1 expression levels in both Aβ_1–42_ mice and Aβ_1–42_ + FTS mice (F (2, 42) = 21.42, *p* < 0.0001, Aβ_1–42_ vs. Aβ_1–42_+ Gal‐3‐AAV: *p* = 0.0001, Aβ_1–42_ vs. Aβ_1–42_ + anisomycin: *p* = 0.0004, Aβ_1–42_ + FTS vs. Aβ_1–42_ + FTS + Gal‐3‐AAV: *p* = 0.0021, Aβ_1–42_ + FTS vs. Aβ_1–42_ + FTS + anisomycin: *p* = 0.0026). These results indicate that FTS may reduce the expression of PS1 through inhibiting the Gal‐3–JNK pathway, which is related to Aβ production. In fact, the administration of anisomycin increased Aβ expression in Aβ_1–42_ mice and Aβ_1–42_ + FTS mice (F (1, 28) = 41.49, *p* < 0.0001; Aβ_1–42_ vs. Aβ_1–42_ + anisomycin: *p* = 0.0149; Aβ_1–42_ + FTS vs. Aβ_1–42_ + FTS + anisomycin: *p* = 0.0004; *n* = 8 mice per group; two‐way ANOVA, followed by Sidak's test; Figure 5E), which was consistent with the change in PS1.

In addition to increasing Aβ production, decreasing Aβ degradation can also increase Aβ oligomerization and accumulation. Neprilysin (NEP) has recently been shown to degrade Aβ in vivo [52], and insulin‐degrading enzyme (IDE) can degrade both Aβ_40_ and Aβ_42_ peptides and cleave Aβ at multiple sites [53]. Transthyretin (TTR) is known to prevent the aggregation of LMW Aβ to generate HMW Aβ [54]. As shown in Figure 5F,H,J, the expression levels of NEP (*t* = 3.830, df = 14, *p* = 0.0018, unpaired Student's *t* test; Figure 5F), IDE (*t* = 3.742, df = 14, *p* = 0.0022, unpaired Student's *t* test; Figure 5H), and TTR (*t* = 4.930, df = 14, *p* = 0.0002, unpaired Student's *t* test; Figure 5J) were reduced in Aβ_1–42_ mice. Interestingly, FTS treatment upregulated the expression levels of NEP (F (1, 28) = 6.257, *p* = 0.0185, Aβ_1–42_ vs. Aβ_1–42_ + FTS: *p* = 0.0025; *n* = 8 mice per group, two‐way ANOVA, followed by Sidak's test; Figure 5G), IDE (F (1, 28) = 6.154, *p* = 0.0194, Aβ_1–42_ vs. Aβ_1–42_ + FTS: *p* = 0.0007; *n* = 8 mice per group, two‐way ANOVA, followed by Sidak's test; Figure 5I) and TTR (F (1, 28) = 13.73, *p* = 0.0009, Aβ_1–42_ vs. Aβ_1–42_ + FTS: *p* = 0.0004; *n* = 8 mice per group, two‐way ANOVA, followed by Sidak's test; Figure 5K), effects that were reversed by Gal‐3 overexpression (NEP: F (1, 28) = 13.07, *p* = 0.0012, Aβ_1–42_ + FTS vs. Aβ_1–42_ + FTS + Gal‐3‐AAV: *p* = 0.0003; IDE: F (1, 28) = 8.084, *p* = 0.0082, Aβ_1–42_ + FTS vs. Aβ_1–42_ + FTS + Gal‐3‐AAV: *p* = 0.0003; TTR: F (1, 28) = 10.96, *p* = 0.0026, Aβ_1–42_ + FTS vs. Aβ_1–42_ + FTS + Gal‐3‐AAV: *p* = 0.0009). However, Gal‐3 overexpression had no effect on the expression levels of these enzymes in Aβ_1–42_ mice (*p =* 0.9092). These results indicate that FTS can increase the expression levels of enzymes that degrade Aβ through the inhibition of Gal‐3 in Aβ_1–42_ mice.

### 3.6 FTS Improves the Cognition of Aβ_1–42_ Mice, Which is Reversed by Gal‐3 Overexpression

**FIGURE 6:**
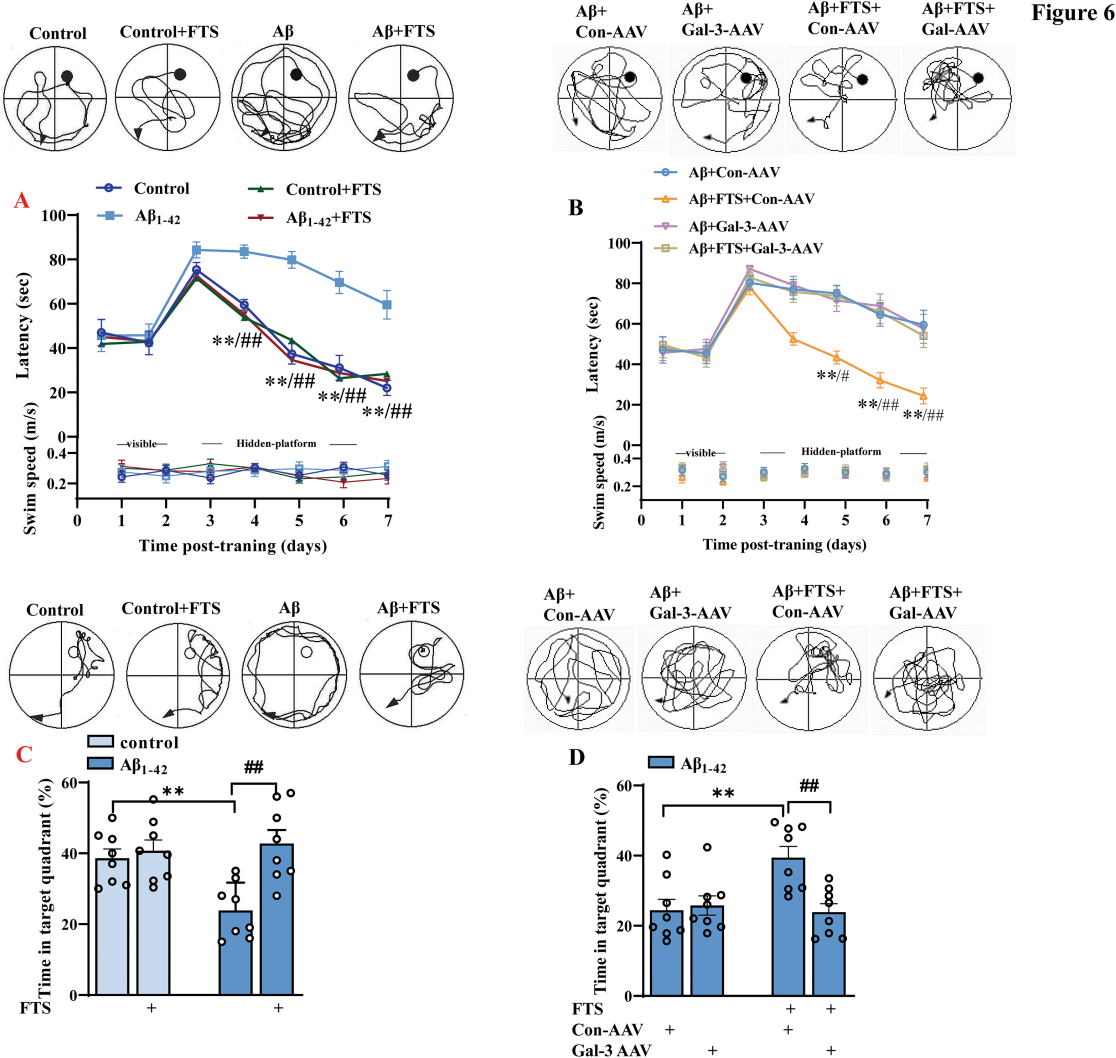
FTS improved cognition in Aβ_1–42_ mice, which was reversed by Gal‐3 overexpression. (A) Latency times to reach the visible and hidden platforms in the MWM test in control and Aβ_1–42_ mice with or without FTS. Representative images of swimming paths in the hidden platform test (Days 3–7) in different groups at 6 days, which are representative. Significant differences were observed in the search times and tracks (upper panels). Black circles: Position of the platform. **p < 0.01 vs. control mice, ##*p* < 0.01 vs. Aβ_1–42_ mice. (B) Latency times to reach the visible and hidden platform in the MWM test in Aβ_1–42_ mice with or without FTS and Gal‐3‐AAV treatment. Representative images of swimming paths in the hidden platform test (Days 3–7) in different groups at 6 days, which are representative. Significant differences were observed in the search times and tracks (upper panels). Black circles: Position of the platform. **p < 0.01 vs. Aβ_1–42_ mice, #*p* < 0.05, ##*p* < 0.01 vs. Aβ_1–42_ mice treated with FTS. (C) Percentages of swimming time (%) in the platform quadrants in the MWM test in control and Aβ_1–42_ mice treated with or without FTS. Tracings of typical swimming patterns in the probe task (upper panels). **p < 0.01 vs. control mice, ^##^ *p* < 0.01 vs. Aβ_1–42_ mice. (D) Percentages of swimming time (%) in the platform quadrants in the MWM test in Aβ_1–42_ mice with or without FTS and Gal‐3‐AAV treatment. Tracings of typical swimming patterns in the probe task (upper panels). **p < 0.01 vs. Aβ_1–42_ mice; ##p < 0.01 vs. Aβ_1–42 m_ mice treated with FTS.

Then, we carried out a MWM test to evaluate the influence of FTS on the condition of Aβ_1–42_ mice. As shown in Figure 6A, the latency to find the visible platform was comparable between control mice and Aβ_1–42_ mice (F (3, 28) = 0.07299, *p* = 0.9740, control vs. Aβ_1–42_: *p* > 0.9999; upper), and there was no significant difference in swimming speed during training days between control mice and Aβ_1–42_ mice (F (3, 28) = 0.08749, *p* = 0.9663; Figure 6A, bottom). Compared with control mice, Aβ_1–42_ mice presented impaired spatial cognitive function, which manifested as a longer time to find the hidden platform (F (3, 28) = 17.11, *p* < 0.0001; Day 4: *p* = 0.0036, Day 5: *p* < 0.0001, Day 6: *p* < 0.0001, Day 7: *p* < 0.0001; *n* = 8, repeated‐measures ANOVA; Figure 6A, upper) and a shorter swimming time in the target quadrant (F (1, 28) = 4.205, *p* = 0.0498, control vs. Aβ_1–42_: *p* = 0.0044; *n* = 8 mice per group, two‐way ANOVA, followed by Sidak's test; Figure 6C). Furthermore, FTS treatment improved the spatial memory of Aβ_1–42_ mice, as shown by the latency to reach the platform (F (1, 28) = 11.76, *p* = 0.0019; Aβ_1–42_ vs. Aβ_1–42_ + FTS: Day 4: *p* = 0.0002, Day 5: *p* < 0.0001, Day 6: *p* < 0.0001, Day 7: *p* < 0.0001; *n* = 8; repeated‐measures ANOVA; Figure 6A), as well as the time spent in the target quadrant (F (1, 28) = 11.44, *p* = 0.0021; Aβ_1–42_ vs. Aβ_1–42_ + FTS: *p* = 0.0004; *n* = 8 mice per group, two‐way ANOVA, followed by Sidak's test; Figure 6C). Gal‐3‐AAV significantly compromised this improvement (latency: F (1, 28) = 9.153, *p* = 0.0053, Aβ_1–42_ + FTS vs. Aβ_1–42_ + FTS + Gal‐3‐AAV: Day 5: *p* = 0.0101, Day 6: *p* < 0.0001, Day 7: *p* = 0.0006, Figure 6B; time in the target quadrant: F (1, 28) = 6.084, *p* = 0.0065, Aβ_1–42_ + FTS vs. Aβ_1–42_ + FTS + Gal‐3‐AAV: *p* = 0.0036, Figure 6D).

## 4 Conclusion and Discussion

To our knowledge, the present study is the first to report the significant protective effects of the Ras inhibitor FTS on neuroinflammation in AD through the targeting of Gal‐3 via multiple pathways, which provides a promising target and an effective way to treat AD.

### 4.1 FTS Regulates Neuroinflammation via Gal‐3 by Directly Inhibiting Microglial Activation and Indirectly Reducing Aβ Pathology to Inhibit Microglial Activation

Gal‐3 has been reported to play a crucial role in the neuroinflammatory response by mediating microglial activation via the IFN‐γ and Janus kinase (JAK)/signal transducer and activator of transcription (STAT) pathways [55]. Our results showed that FTS treatment relieved the inflammatory response in Aβ_1–42_ mice (reducing the release of inflammatory molecules and inhibiting microglial activation, Figure 1A–I) through the inhibition of Gal‐3.

TLR‐stimulated signaling cascades act to upregulate the expression of proinflammatory cytokines and chemokines, NO synthase, and other antimicrobial peptides that directly destroy microbial pathogens, as reviewed by [56]. As an endogenous paracrine TLR4 ligand, Gal‐3 can bind to microglial TLR4, triggering the proinflammatory response under acute neuroinflammatory conditions. The expression and release of Gal‐3 are significantly increased by the proinflammatory cytokine IFN‐γ in microglia [55]. Given the direct relationship between Gal‐3 and inflammation, we speculate that FTS can downregulate Gal‐3 expression to inhibit microglial activation, which inhibits inflammation in the brains of Aβ_1–42_ mice. This study focused mainly on the inflammatory response induced by Aβ pathology, which was related to Gal‐3 overexpression in Aβ_1–42_ mice. Remarkably, in patients with AD, Aβ senile plaques are often found close to reactive microglia [57, 58]. In vitro, Aβ_1–42_ has been shown to activate microglia via CD36 and the TLR2–TLR6 heterodimer, and elevated levels of TNF‐α and iNOS have also been reported in Aβ‐treated primary rat microglia [59]. Disaggregation of AβOs can decrease Aβ‐induced inflammation and rescue cognitive deficits in APP/PS1 mice [60]. In our study, AβOs and their accumulation significantly increased (Figure 3A–F), effects that were reduced by FTS treatment and reversed by Gal‐3 overexpression. In addition, FTS promoted the production of Aβ via the Gal‐3–JNK pathway (Figure 4A–E) and reduced the degradation of Aβ by increasing the number of degrading enzymes in a Gal‐3‐dependent manner (Figure 5A–F). As a result, we speculated that FTS regulated the inflammatory response in Aβ_1–42_ mice by directly inhibiting microglial activation and indirectly reducing Aβ pathology, both by targeting Gal‐3.

### 4.2 FTS Regulates the Intracellular Distribution of Gal‐3 to Inhibit Inflammation in AD

Endogenous Gal‐3 is found mainly in the cytoplasm, and its expression is also observed on the cell membrane and in the nucleus; Gal‐3 can also be released into the extracellular space upon stimulation (such as with LPS and IFN‐γ) [61]. The different subcellular localizations of Gal‐3 together with its possible posttranslational modifications are likely to affect the function of Gal‐3 and explain the conflicting findings about Gal‐3, for example, its pro‐ versus antiapoptotic effects [62] and pro‐ versus anti‐inflammatory effects [16]. Recent studies have focused on the role of secreted Gal‐3 in inflammation‐associated diseases [63]. There is evidence that extracellular Gal‐3 is able to activate immune and inflammatory signaling pathways through the phosphorylation of STAT1, STAT3, STAT5, and JAK2 [16]. The extracellular biological activities of Gal‐3 are dependent mainly on binding cell surface and extracellular matrix glycan‐related ligands [64]. Increased Gal‐3 expression in the extracellular space has already been confirmed in some human diseases, including tumors, neurodegenerative diseases, and cardiovascular diseases [65]. A clear FTS‐induced redistribution of Gal‐3 from the cell membrane was observed in our study. We found that FTS treatment reduced the membrane expression (Figure 2C) of Gal‐3 but increased its cytoplasmic expression (Figure 2D), interestingly decreasing the total expression (Figure 2B) and its release (Figure 2A), consistent with the findings of another study [25]: Gal‐3 in ARO cancer cells was found to be localized in the cytoplasm and on the cell membrane before FTS treatment, but FTS treatment significantly increased the cytoplasmic Gal‐3 content. We speculate that in our study, in the hippocampus of AD model mice, FTS dislodges Gal‐3 from the plasma membrane and cytosolic Gal‐3 is then degraded, which induces a decreased level of total Gal‐3 and inhibits its release. However, the specific molecular mechanisms involved should be further elucidated.

Taken together, our study revealed that FTS may directly reduce the expression levels of TLR4 and CD14 by targeting Gal‐3, alleviate the neuroinflammatory response, or reduce the production of Aβ via the inhibition of the Gal‐3–JNK–PS1 pathway and increase the levels of enzymes that degrade Aβ through the inhibition of Gal‐3 content; in this way, FTS indirectly downregulates TLR2, TLR4, and CD14, which are stimulated by Aβ; the direct and indirect decrease in TLRs induces improved neuroinflammation in Aβ_1–42_ mice.

## Author Contributions

Qing Qiu, Cuicui Li, Xiaoli Zhao, and Menting Yang performed the research and analyzed the data. Haiying Liang and Shushu Ding edited the manuscript. Tingting Chen designed the research and wrote the manuscript.

## Disclosure

The authors have nothing to report.

## Conflicts of Interest

The authors declare no conflicts of interest.

## Supporting information


**Figure S1.** The dose‐dependent effects of FTS on the inflammatory factors in Aβ_1–42_ mice, including IL‐1β, IL‐6, TNF‐α, and NO, FTS (1, 3, and 10 mg/kg) was administered once daily. **p < 0.01 vs. Aβ_1–42_ mice.

Data S1.

## Data Availability

The data that support the findings of this study are available from the corresponding author upon reasonable request.

## References

[1] L. Zhong , X. F. Chen , T. Wang , et al., “Soluble TREM2 Induces Inflammatory Responses and Enhances Microglial Survival,” Journal of Experimental Medicine 214, no. 3 (2017): 597–607. 28209725 10.1084/jem.20160844 PMC5339672

[2] W. Xie , B. Cao , H. Zhu , et al., “Orally Bioavailable Prodrugs of Psi‐GSH: A Potential Treatment for Alzheimer's Disease,” Journal of Medicinal Chemistry 65, no. 21 (2022): 14441–14455. 36353871 10.1021/acs.jmedchem.2c00779 PMC9662183

[3] A. Shastri , D. M. Bonifati , and U. Kishore , “Innate Immunity and Neuroinflammation,” Mediators of Inflammation 2013 (2013): 342931. 23843682 10.1155/2013/342931 PMC3697414

[4] Z. Yin , D. Raj , N. Saiepour , et al., “Immune Hyperreactivity of Abeta Plaque‐Associated Microglia in Alzheimer's Disease,” Neurobiology of Aging 55 (2017): 115–122. 28434692 10.1016/j.neurobiolaging.2017.03.021

[5] J. Cohen , A. Mathew , K. D. Dourvetakis , et al., “Recent Research Trends in Neuroinflammatory and Neurodegenerative Disorders,” Cells 13, no. 6 (2024): 511. 38534355 10.3390/cells13060511 PMC10969521

[6] Q. Alam , M. Z. Alam , G. Mushtaq , et al., “Inflammatory Process in Alzheimer's and Parkinson's Diseases: Central Role of Cytokines,” Current Pharmaceutical Design 22, no. 5 (2016): 541–548. 26601965 10.2174/1381612822666151125000300

[7] M. P. Kummer , C. Hulsmann , M. Hermes , D. Axt , and M. T. Heneka , “Nitric Oxide Decreases the Enzymatic Activity of Insulin Degrading Enzyme in APP/PS1 Mice,” Journal of Neuroimmune Pharmacology 7, no. 1 (2012): 165–172. 22227962 10.1007/s11481-011-9339-7

[8] F. Leng and P. Edison , “Neuroinflammation and Microglial Activation in Alzheimer Disease: Where Do We Go From Here?,” Nature Reviews. Neurology 17, no. 3 (2021): 157–172. 33318676 10.1038/s41582-020-00435-y

[9] T. Yazar , H. Olgun Yazar , and M. Cihan , “Evaluation of Serum Galectin‐3 Levels at Alzheimer Patients by Stages: A Preliminary Report,” Acta Neurologica Belgica 121, no. 4 (2021): 949–954. 32852752 10.1007/s13760-020-01477-1

[10] C. F. Brewer , M. C. Miceli , and L. G. Baum , “Clusters, Bundles, Arrays and Lattices: Novel Mechanisms for Lectin‐Saccharide‐Mediated Cellular Interactions,” Current Opinion in Structural Biology 12, no. 5 (2002): 616–623. 12464313 10.1016/s0959-440x(02)00364-0

[11] Q. Zhao , M. Barclay , J. Hilkens , et al., “Interaction Between Circulating Galectin‐3 and Cancer‐Associated MUC1 Enhances Tumour Cell Homotypic Aggregation and Prevents Anoikis,” Molecular Cancer 9 (2010): 154. 20565834 10.1186/1476-4598-9-154 PMC2911446

[12] M. Hoffmann , M. R. Hayes , J. Pietruszka , and L. Elling , “Synthesis of the Thomsen‐Friedenreich‐Antigen (TF‐Antigen) and Binding of Galectin‐3 to TF‐Antigen Presenting Neo‐Glycoproteins,” Glycoconjugate Journal 37, no. 4 (2020): 457–470. 32367478 10.1007/s10719-020-09926-y PMC7329766

[13] G. A. Rabinovich , L. G. Baum , N. Tinari , et al., “Galectins and Their Ligands: Amplifiers, Silencers or Tuners of the Inflammatory Response?,” Trends in Immunology 23, no. 6 (2002): 313–320. 12072371 10.1016/s1471-4906(02)02232-9

[14] T. Shimura , Y. Takenaka , S. Tsutsumi , V. Hogan , A. Kikuchi , and A. Raz , “Galectin‐3, a Novel Binding Partner of Beta‐Catenin,” Cancer Research 64, no. 18 (2004): 6363–6367. 15374939 10.1158/0008-5472.CAN-04-1816

[15] Y. Li , M. Komai‐Koma , D. S. Gilchrist , et al., “Galectin‐3 Is a Negative Regulator of Lipopolysaccharide‐Mediated Inflammation,” Journal of Immunology 181, no. 4 (2008): 2781–2789. 10.4049/jimmunol.181.4.2781 18684969

[16] S. B. Jeon , H. J. Yoon , C. Y. Chang , H. S. Koh , S. H. Jeon , and E. J. Park , “Galectin‐3 Exerts Cytokine‐Like Regulatory Actions Through the JAK‐STAT Pathway,” Journal of Immunology 185, no. 11 (2010): 7037–7046. 10.4049/jimmunol.1000154 20980634

[17] K. Satoh , M. Niwa , N. H. Binh , et al., “Increase of Galectin‐3 Expression in Microglia by Hyperthermia in Delayed Neuronal Death of Hippocampal CA1 Following Transient Forebrain Ischemia,” Neuroscience Letters 504, no. 3 (2011): 199–203. 21945545 10.1016/j.neulet.2011.09.015

[18] M. Lalancette‐Hebert , G. Gowing , A. Simard , Y. C. Weng , and J. Kriz , “Selective Ablation of Proliferating Microglial Cells Exacerbates Ischemic Injury in the Brain,” Journal of Neuroscience 27, no. 10 (2007): 2596–2605. 17344397 10.1523/JNEUROSCI.5360-06.2007 PMC6672496

[19] U. V. Wesley , R. Vemuganti , E. R. Ayvaci , and R. J. Dempsey , “Galectin‐3 Enhances Angiogenic and Migratory Potential of Microglial Cells via Modulation of Integrin Linked Kinase Signaling,” Brain Research 1496 (2013): 1–9. 23246924 10.1016/j.brainres.2012.12.008 PMC4084961

[20] P. Chowdari Gurram , S. Satarker , and M. Nampoothiri , “Recent Advances in the Molecular Signaling Pathways of Substance P in Alzheimer's Disease: Link to Neuroinflammation Associated With Toll‐Like Receptors,” Biochemical and Biophysical Research Communications 733 (2024): 150597. 39197195 10.1016/j.bbrc.2024.150597

[21] G. E. Landreth and E. G. Reed‐Geaghan , “Toll‐Like Receptors in Alzheimer's Disease,” Current Topics in Microbiology and Immunology 336 (2009): 137–153. 19688332 10.1007/978-3-642-00549-7_8 PMC3032986

[22] S. Erlich , P. Tal‐Or , R. Liebling , et al., “Ras Inhibition Results in Growth Arrest and Death of Androgen‐Dependent and Androgen‐Independent Prostate Cancer Cells,” Biochemical Pharmacology 72, no. 4 (2006): 427–436. 16780807 10.1016/j.bcp.2006.05.007

[23] R. Haklai , G. Elad‐Sfadia , Y. Egozi , and Y. Kloog , “Orally Administered FTS (Salirasib) Inhibits Human Pancreatic Tumor Growth in Nude Mice,” Cancer Chemotherapy and Pharmacology 61, no. 1 (2008): 89–96. 17909812 10.1007/s00280-007-0451-6

[24] R. Shalom‐Feuerstein , S. J. Plowman , B. Rotblat , et al., “K‐Ras Nanoclustering Is Subverted by Overexpression of the Scaffold Protein Galectin‐3,” Cancer Research 68, no. 16 (2008): 6608–6616. 18701484 10.1158/0008-5472.CAN-08-1117 PMC2587079

[25] R. Levy , M. Grafi‐Cohen , Z. Kraiem , and Y. Kloog , “Galectin‐3 Promotes Chronic Activation of K‐Ras and Differentiation Block in Malignant Thyroid Carcinomas,” Molecular Cancer Therapeutics 9, no. 8 (2010): 2208–2219. 20682656 10.1158/1535-7163.MCT-10-0262

[26] Y. Wang , T. Chen , Z. Yuan , et al., “Ras Inhibitor S‐Trans, Trans‐Farnesylthiosalicylic Acid Enhances Spatial Memory and Hippocampal Long‐Term Potentiation via Up‐Regulation of NMDA Receptor,” Neuropharmacology 139 (2018): 257–267. 29578035 10.1016/j.neuropharm.2018.03.026

[27] X. Wang , Y. Wang , Y. Zhu , L. Yan , and L. Zhao , “Neuroprotective Effect of S‐Trans, Trans‐Farnesylthiosalicylic Acid via Inhibition of RAS/ERK Pathway for the Treatment of Alzheimer's Disease,” Drug Design, Development and Therapy 13 (2019): 4053–4063. 31819374 10.2147/DDDT.S233283 PMC6890185

[28] E. Shohami , I. Yatsiv , A. Alexandrovich , et al., “The Ras Inhibitor S‐Trans, Trans‐Farnesylthiosalicylic Acid Exerts Long‐Lasting Neuroprotection in a Mouse Closed Head Injury Model,” Journal of Cerebral Blood Flow and Metabolism 23, no. 6 (2003): 728–738. 12796721 10.1097/01.WCB.0000067704.86573.83

[29] C. Saelee , J. Hanthamrongwit , P. T. Soe , et al., “Toll‐Like Receptor‐Mediated Innate Immune Responses by Recognition of the Recombinant Dormancy‐Associated *Mycobacterium tuberculosis* Proteins Rv2659c and Rv1738,” PLoS One 17, no. 9 (2022): e0273517. 36048884 10.1371/journal.pone.0273517 PMC9436120

[30] T. Kameyama , T. Nabeshima , and T. Kozawa , “The Antagonistic Effects of Naloxone on Cycloheximide and Anisomycin‐Induced Amnesia,” Pharmacology, Biochemistry, and Behavior 25, no. 3 (1986): 567–572. 3774821 10.1016/0091-3057(86)90142-5

[31] R. Stennett , M. Katz , V. Jackson‐Lewis , S. Fahn , and J. L. Cadet , “The Protein Synthesis Inhibitor, Anisomycin, Causes Exacerbation of the Iminodipropionitrile‐Induced Spasmodic Dyskinetic Syndrome in Rats,” Pharmacology, Biochemistry, and Behavior 32, no. 4 (1989): 1003–1008. 2477862 10.1016/0091-3057(89)90073-7

[32] M. J. Robinson and K. B. Franklin , “Effects of Anisomycin on Consolidation and Reconsolidation of a Morphine‐Conditioned Place Preference,” Behavioural Brain Research 178, no. 1 (2007): 146–153. 17239969 10.1016/j.bbr.2006.12.013

[33] C. Wang , T. Chen , G. Li , L. Zhou , S. Sha , and L. Chen , “Simvastatin Prevents Beta‐Amyloid(25‐35)‐impaired Neurogenesis in Hippocampal Dentate Gyrus Through alpha7nAChR‐Dependent Cascading PI3K‐Akt and Increasing BDNF via Reduction of Farnesyl Pyrophosphate,” Neuropharmacology 97 (2015): 122–132. 26051402 10.1016/j.neuropharm.2015.05.020

[34] R. L. Richardson , E. M. Kim , R. A. Shephard , T. Gardiner , J. Cleary , and E. O'Hare , “Behavioural and Histopathological Analyses of Ibuprofen Treatment on the Effect of Aggregated Abeta(1–42) Injections in the Rat,” Brain Research 954, no. 1 (2002): 1–10. 12393227 10.1016/s0006-8993(02)03006-8

[35] S. H. Choi and R. E. Tanzi , “Adult neurogenesis in Alzheimer's Disease,” Hippocampus 33, no. 4 (2023): 307–321. 36748337 10.1002/hipo.23504

[36] Y. Qian , J. Yin , J. Hong , et al., “Neuronal Seipin Knockout Facilitates Abeta‐Induced Neuroinflammation and Neurotoxicity via Reduction of PPARgamma in Hippocampus of Mouse,” Journal of Neuroinflammation 13, no. 1 (2016): 145. 27287266 10.1186/s12974-016-0598-3 PMC4902906

[37] J. Deng , X. Feng , L. Zhou , et al., “Heterophyllin B, a Cyclopeptide From Pseudostellaria Heterophylla, Improves Memory via Immunomodulation and Neurite Regeneration in i.c.v.Abeta‐Induced Mice,” Food Research International 158 (2022): 111576. 35840261 10.1016/j.foodres.2022.111576

[38] C. Cai , L. Wang , S. Li , et al., “Ras Inhibitor Lonafarnib Rescues Structural and Functional Impairments of Synapses of Abeta(1‐42) Mice via alpha7nAChR‐Dependent BDNF Upregulation,” Journal of Neuroscience 42, no. 31 (2022): 6090–6107. 35760529 10.1523/JNEUROSCI.1989-21.2022 PMC9351638

[39] T. Chen , C. Wang , S. Sha , L. Zhou , L. Chen , and L. Chen , “Simvastatin Enhances Spatial Memory and Long‐Term Potentiation in Hippocampal CA1 via Upregulation of alpha7 Nicotinic Acetylcholine Receptor,” Molecular Neurobiology 53, no. 6 (2016): 4060–4072. 26198568 10.1007/s12035-015-9344-6

[40] W. Feng , Y. Zhang , P. Sun , and M. Xiao , “Acquired Immunity and Alzheimer's Disease,” Journal of Biomedical Research 37, no. 1 (2022): 15–29. 36165328 10.7555/JBR.36.20220083 PMC9898041

[41] N. Esen and T. Kielian , “Central Role for MyD88 in the Responses of Microglia to Pathogen‐Associated Molecular Patterns,” Journal of Immunology 176, no. 11 (2006): 6802–6811. 10.4049/jimmunol.176.11.6802 PMC2440502 16709840

[42] M. Letiembre , W. Hao , Y. Liu , et al., “Innate Immune Receptor Expression in Normal Brain Aging,” Neuroscience 146, no. 1 (2007): 248–254. 17293054 10.1016/j.neuroscience.2007.01.004

[43] S. Walter , M. Letiembre , Y. Liu , et al., “Role of the Toll‐Like Receptor 4 in Neuroinflammation in Alzheimer's Disease,” Cellular Physiology and Biochemistry 20, no. 6 (2007): 947–956. 17982277 10.1159/000110455

[44] E. Okun , K. J. Griffioen , and M. P. Mattson , “Toll‐Like Receptor Signaling in Neural Plasticity and Disease,” Trends in Neurosciences 34, no. 5 (2011): 269–281. 21419501 10.1016/j.tins.2011.02.005 PMC3095763

[45] Q. Wang , X. Yu , L. Li , and J. Zheng , “Inhibition of Amyloid‐Beta Aggregation in Alzheimer's Disease,” Current Pharmaceutical Design 20, no. 8 (2014): 1223–1243. 23713775 10.2174/13816128113199990068

[46] B. Maity , S. Kameyama , J. Tian , et al., “Fusion of Amyloid Beta With Ferritin Yields an Isolated Oligomeric Beta‐Sheet‐Rich Aggregate Inside the Ferritin Cage,” Biomaterials Science 12, no. 9 (2024): 2408–2417. 38511491 10.1039/d4bm00173g

[47] S. Lee , E. J. Fernandez , and T. A. Good , “Role of Aggregation Conditions in Structure, Stability, and Toxicity of Intermediates in the Abeta Fibril Formation Pathway,” Protein Science 16, no. 4 (2007): 723–732. 17327396 10.1110/ps.062514807 PMC2203338

[48] M. Ray and S. C. Lakhotia , “Activated Ras/JNK Driven Dilp8 in Imaginal Discs Adversely Affects Organismal Homeostasis During Early Pupal Stage in Drosophila, a New Checkpoint for Development,” Developmental Dynamics 248, no. 12 (2019): 1211–1231. 31415125 10.1002/dvdy.102

[49] B. De Strooper , P. Saftig , K. Craessaerts , et al., “Deficiency of Presenilin‐1 Inhibits the Normal Cleavage of Amyloid Precursor Protein,” Nature 391, no. 6665 (1998): 387–390. 9450754 10.1038/34910

[50] J. Y. Hur , “Gamma‐Secretase in Alzheimer's Disease,” Experimental & Molecular Medicine 54, no. 4 (2022): 433–446. 35396575 10.1038/s12276-022-00754-8 PMC9076685

[51] S. Lee and H. K. Das , “Inhibition of Basal Activity of c‐Jun‐NH2‐Terminal Kinase (JNK) Represses the Expression of Presenilin‐1 by a p53‐Dependent Mechanism,” Brain Research 1207 (2008): 19–31. 18374905 10.1016/j.brainres.2008.02.016

[52] E. A. Eckman , M. Watson , L. Marlow , K. Sambamurti , and C. B. Eckman , “Alzheimer's Disease Beta‐Amyloid Peptide Is Increased in Mice Deficient in Endothelin‐Converting Enzyme,” Journal of Biological Chemistry 278, no. 4 (2003): 2081–2084. 12464614 10.1074/jbc.C200642200

[53] W. Farris , S. Mansourian , Y. Chang , et al., “Insulin‐Degrading Enzyme Regulates the Levels of Insulin, Amyloid Beta‐Protein, and the Beta‐Amyloid Precursor Protein Intracellular Domain In Vivo,” Proceedings of the National Academy of Sciences of the United States of America 100, no. 7 (2003): 4162–4167. 12634421 10.1073/pnas.0230450100 PMC153065

[54] X. Li , X. Zhang , A. R. Ladiwala , et al., “Mechanisms of Transthyretin Inhibition of Beta‐Amyloid Aggregation In Vitro,” Journal of Neuroscience 33, no. 50 (2013): 19423–19433. 24336709 10.1523/JNEUROSCI.2561-13.2013 PMC3858619

[55] Y. Tan , Y. Zheng , D. Xu , Z. Sun , H. Yang , and Q. Yin , “Galectin‐3: A Key Player in Microglia‐Mediated Neuroinflammation and Alzheimer's Disease,” Cell & Bioscience 11, no. 1 (2021): 78. 33906678 10.1186/s13578-021-00592-7 PMC8077955

[56] J. Han and R. J. Ulevitch , “Limiting Inflammatory Responses During Activation of Innate Immunity,” Nature Immunology 6, no. 12 (2005): 1198–1205. 16369559 10.1038/ni1274

[57] M. T. He , J. H. Kim , and E. J. Cho , “Co‐Treatment With the Seed of *Carthamus tinctorius* L. and the Aerial Part of Taraxacum Coreanum Synergistically Suppresses Abeta(25‐35)‐Induced Neurotoxicity by Altering APP Processing,” Food Science & Nutrition 12, no. 3 (2024): 1573–1580. 38455162 10.1002/fsn3.3768 PMC10916591

[58] A. F. Carpenter , P. W. Carpenter , and W. R. Markesbery , “Morphometric Analysis of Microglia in Alzheimer's Disease,” Journal of Neuropathology and Experimental Neurology 52, no. 6 (1993): 601–608. 8229079 10.1097/00005072-199311000-00007

[59] S. T. Dheen , Y. Jun , Z. Yan , S. S. Tay , and E. A. Ling , “Retinoic Acid Inhibits Expression of TNF‐Alpha and iNOS in Activated Rat Microglia,” Glia 50, no. 1 (2005): 21–31. 15602748 10.1002/glia.20153

[60] H. Y. Kim , H. V. Kim , S. Jo , et al., “EPPS Rescues Hippocampus‐Dependent Cognitive Deficits in APP/PS1 Mice by Disaggregation of Amyloid‐Beta Oligomers and Plaques,” Nature Communications 6 (2015): 8997. 10.1038/ncomms9997 PMC4686862 26646366

[61] M. A. Burguillos , M. Svensson , T. Schulte , et al., “Microglia‐Secreted Galectin‐3 Acts as a Toll‐Like Receptor 4 Ligand and Contributes to Microglial Activation,” Cell Reports 10, no. 9 (2015): 1626–1638. 25753426 10.1016/j.celrep.2015.02.012

[62] S. Nakahara , N. Oka , and A. Raz , “On the Role of Galectin‐3 in Cancer Apoptosis,” Apoptosis 10, no. 2 (2005): 267–275. 15843888 10.1007/s10495-005-0801-y

[63] S. Sato , C. St‐Pierre , P. Bhaumik , and J. Nieminen , “Galectins in Innate Immunity: Dual Functions of Host Soluble Beta‐Galactoside‐Binding Lectins as Damage‐Associated Molecular Patterns (DAMPs) and as Receptors for Pathogen‐Associated Molecular Patterns (PAMPs),” Immunological Reviews 230, no. 1 (2009): 172–187. 19594636 10.1111/j.1600-065X.2009.00790.x

[64] J. Ochieng , V. Furtak , and P. Lukyanov , “Extracellular Functions of Galectin‐3,” Glycoconjugate Journal 19, no. 7–9 (2002): 527–535. 14758076 10.1023/B:GLYC.0000014082.99675.2f

[65] X. Zhong , X. Qian , G. Chen , and X. Song , “The Role of Galectin‐3 in Heart Failure and Cardiovascular Disease,” Clinical and Experimental Pharmacology & Physiology 46, no. 3 (2019): 197–203. 30372548 10.1111/1440-1681.13048

