# Supplementary material for: Farnesylthiosalicylic Acid Through Inhibition of Galectin‐3 Improves Neuroinflammation in Alzheimer Disease via Multiple Pathways
Source: CNS Neurosci Ther. 2024 Nov 26;30(11):e70127. doi: 10.1111/cns.70127 (PMC11598744; doi:10.1111/cns.70127)
Supplement: Supplementary file 2 — Data S1. [file CNS-30-e70127-s001.zip › R1-Supplementary Material 1.docx]

*2.4 Morris Water Maze (MWM) test*

A circular, black, plastic pool (120 cm in diameter) is artificially divided into four quadrants and marked on the wall with an entry point for each quadrant. The water temperature was maintained at 23±2℃, and an appropriate amount of white food additives was added into the water. The swim paths were analysed using a computer system with a video camera (AXIS-90 Target/2; Neuroscience). The test is divided into two parts, place navigation and spatial probe test. Place navigation includes visible platform test (days 1-2) and hidden platform test (training days 3-7). In the first two days of training, a cylindrical dark-coloured platform (7 cm in diameter) was placed 0.5 cm above the surface of water. During training days 3-7 (the acquisition‑testing phase), the platform was submerged 1 cm below the surface of water. Mice were allowed to search the platform within 90 s in the pool. The latency to reaching the visible or hidden platform and the swimming distance were measured. During the training, each mouse was placed at one of four quadrants randomly, with its head towards the wall. If the mouse failed to find the platform within 90 s, it was guided to the platform and the test was terminated. Four trials were conducted every day with an interval of 30 min. In spatial probe test (day 8), the retention of spatial reference memory was recorded in a probe trial with the platform being removed from the pool, and the percent time spent in each quadrant was calculated.

*2.5 Western Blotting*

Animals were anesthetized, and the brain was harvested, followed by the separation of hippocampus. The hippocampal tissues or brain slices were homogenized in the lysis buffer containing 50 mM Tris–HCl (pH 7.5), 150 mM NaCl, 5 mM EDTA, 10 mM NaF, 1 mM sodium orthovanadate, 1% Triton X-100, 0.5% sodium deoxycholate, 1 mM phenylmethylsulfonyl fluoride and protease inhibitor cocktail (Complete; Roche, Mannheim, Germany), followed by incubation for 30 min at 4°C. After sonication, the samples were centrifuged at 12000 rpm for 15 min at 4°C, and the supernatant was harvested. The protein concentration was determined with BCA Protein Assay Kit (Pierce Biotechnology Inc., Rockford, IL, United States). Then, proteins of equal amount were mixed with loading buffer and boiled for 5 min.

Proteins of equal amount (20 μg) were separated by SDS-polyacrylamide gel electrophoresis (SDS–PAGE), and then transferred onto polyvinylidene fluoride (PVDF) membrane, which was subsequently incubated with 5% nonfat milk for 60 min at room temperature. After washing thrice, the membrane was incubated overnight at 4°C with primary antibodies. Image J (NIH Image, Bethesda, MD, USA) was used to determine the protein expression which was normalized to the expression of internal control.

*2.6 Tissue fixation and immunofluorescence staining*

Animals were anesthetized with isoflurane and transcardially perfused with 4% paraformaldehyde. Brains were removed and post-fixed overnight in the same solution, Brains were transferred into 20% and 30% sucrose sequentially. After gradient dehydration, serial coronal sections of the hippocampus (40 μm) were obtained in a freezing microtome (Leica, Nussloch, Germany). A total of 50 sections were obtained from each brain and the fifth section of the hippocampus was processed for cell counting. In order to obtain a homogenous representation of the hippocampus, no more than 2 sections were lost during sectioning of a single brain. The sections were treated with 3% normal goat serum, and then incubated overnight at 4°C with antibodies. Positive area and plaques were counted by an investigator who was blind to the grouping. The sections were observed under a microscope (Leica TCS SP8). The number and area of positive puncta were quantified with NIH ImageJ freeware (Wayne Rasband, NIH).

*2.7 Enzyme-linked immunosorbent assay (ELISA)*

The contents of released interleukin-1β (IL-1β), IL-6, tumor necrosis factor-α (TNF-α), nitric oxide (NO) and Gal-3 were detected by enzyme-linked immunosorbent assay (ELISA). The hippocampus was homogenized by sonication in homogenization buffer (50 mM Tris pH 7.5, 300 mM NaCl, 0.1% Triton X-100, 10 mg/mL aprotinin, 0.1 mM benzethonium chloride, 1 mM benzamidine, and 0.1 mM phenylmethylsulfonyl fluoride), followed by centrifugation for 15 min at 12,000 g at 4℃. Briefly, titer plates were coated with anti-IL-1β, IL-6, TNF-α, NO or Gal-3 monoclonal antibody overnight at 4℃. The plates were incubated with IL-1β, IL-6, TNF-α, NO or Gal-3 polyclonal antibody at room temperature. HRP-conjugated anti-IgY rabbit antibody was added, followed by incubation at room temperature. The absorbance was measured at 450 nm with an automated microplate reader. The contents of released IL-1β, IL-6, TNF-α, NO and Gal-3 were expressed as pg/mg total soluble protein. All assays were performed in triplicate.

*2.9 Reverse Transcription-Polymerase Chain Reaction (RT-PCR)*

Total RNA was isolated from the hippocampus with TRIzol reagent (Invitrogen, Camarillo, CA) and reverse-transcribed into cDNA using a Prime Script RT reagent kit (Takara, China) for quantitative PCR (ABI Step One Plus, Foster City, CA) in the presence of fluorescent dye (SYBR Green I; Takara, China). The relative expression of target genes was determined using the 2^−ΔΔct^ method with *GAPDH* as an internal control. The primers used for PCR were as follows: *IL-1β*, 5′-AAGCCTCGTGCTGTCGGACC-3′ (forward) and 5′- TGAGGCCCAAGGCCACAGGT -3′ (reverse); *IL-6*; 5′-CAAAGCCAGAGTCCTTGAGA-3′ (forward) and 5′-GATGGTCTT GGTCCTTAGCC-3′ (reverse); *TNF-α*, 5′-CAAGGGACAAGGCTGCCCCG-3′ (forward) and 5′-GCAGGGGCTCTTGACGGCAG-3′ (reverse); *GAPDH*, 5′-TGGGTGTGAACCACGAG-3′ (forward) and 5′- AAGTTGTCATGGATGACCTT-3′ (reverse).
