# Supplementary material for: Farnesylthiosalicylic Acid Through Inhibition of Galectin‐3 Improves Neuroinflammation in Alzheimer Disease via Multiple Pathways
Source: CNS Neurosci Ther. 2024 Nov 26;30(11):e70127. doi: 10.1111/cns.70127 (PMC11598744; doi:10.1111/cns.70127)

Full unedited gel for Figure 2B

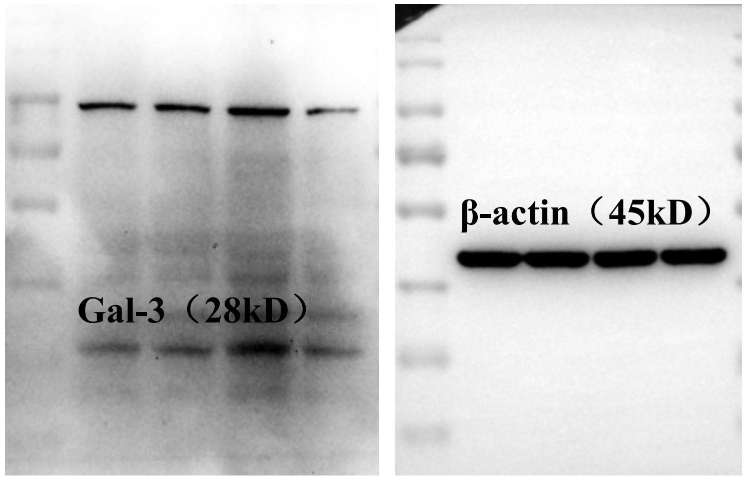

Full unedited gel for Figure 2C

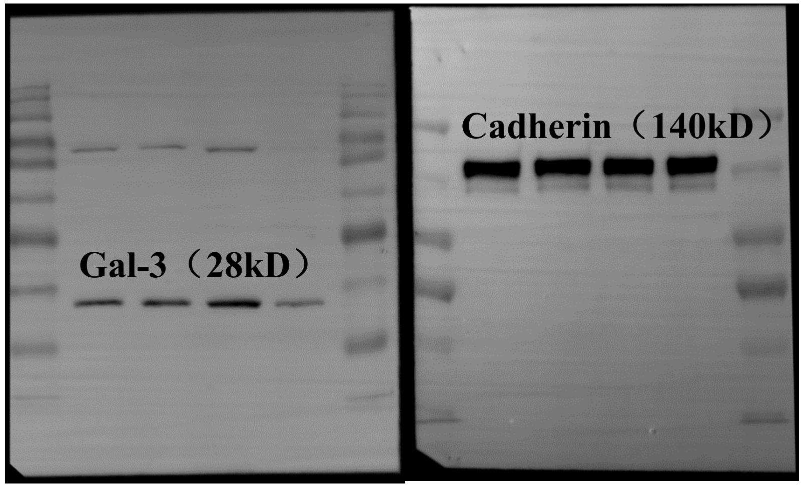

Full unedited gel for Figure 2D

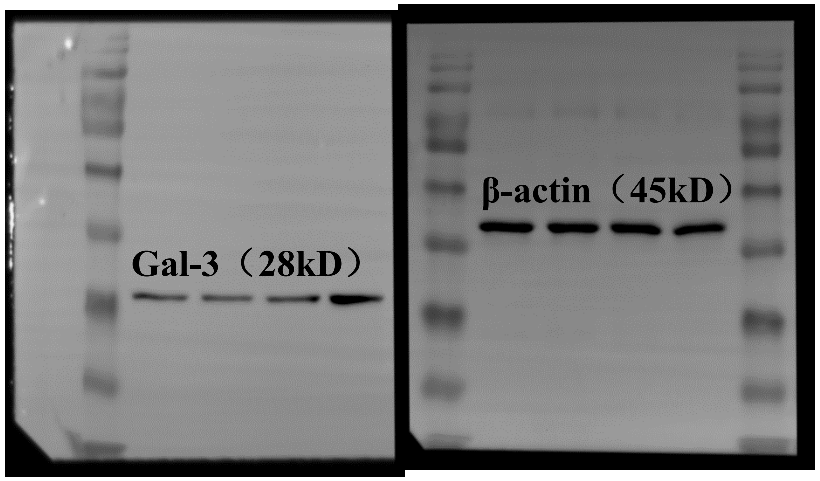

Full unedited gel for Figure 3E

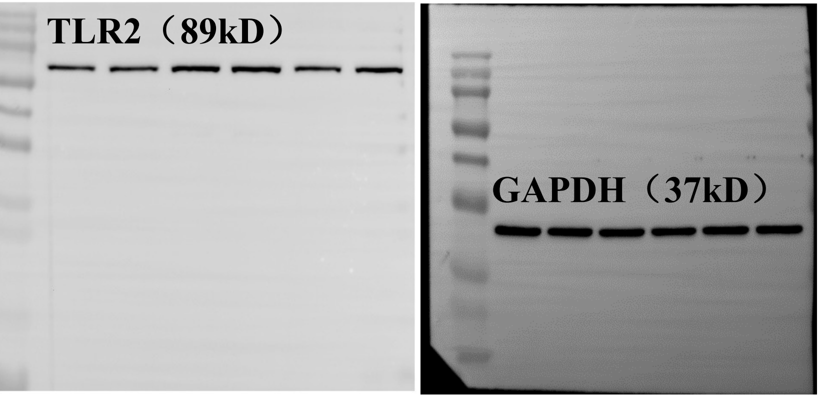

Full unedited gel for Figure 3F

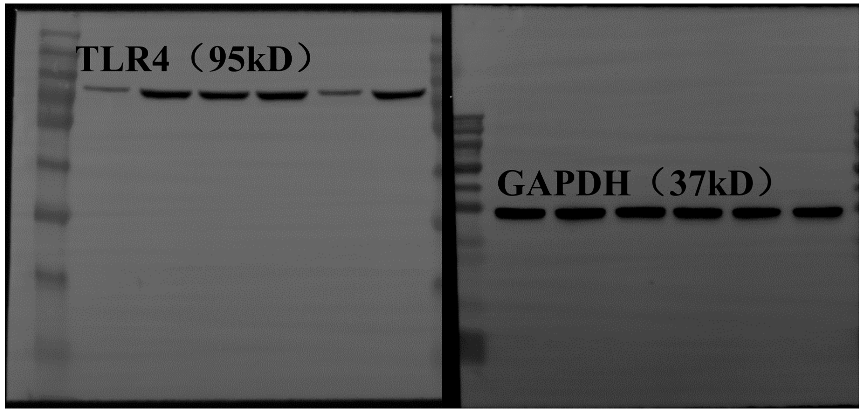

Full unedited gel for Figure 3G

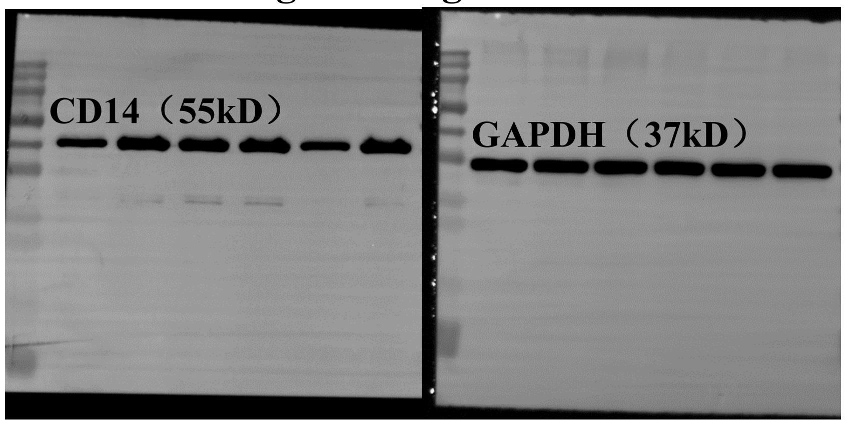

Full unedited gel for Figure 3H

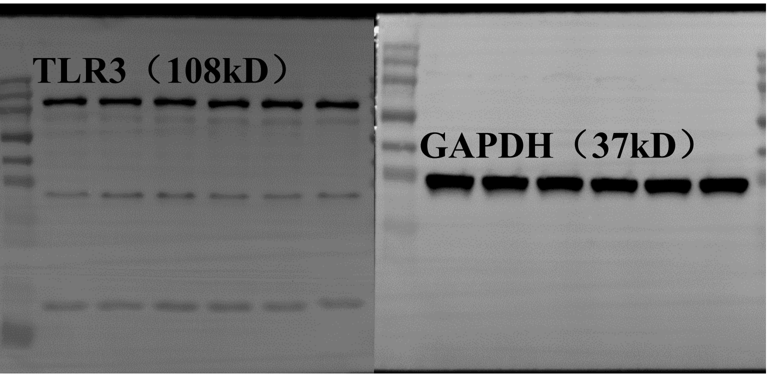

Full unedited gel for Figure 3I

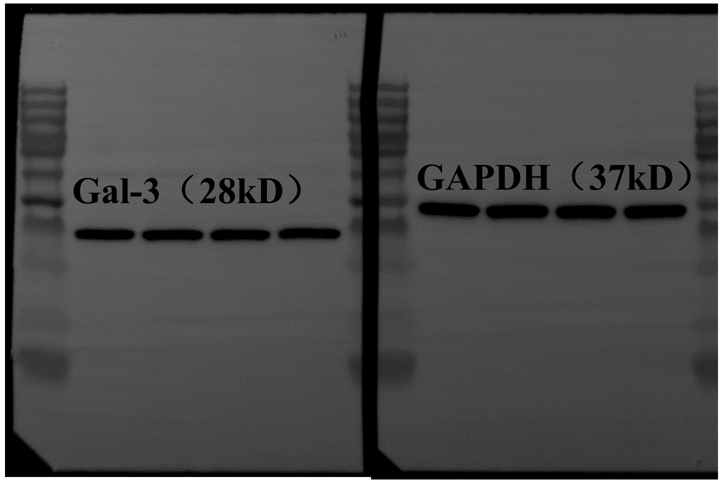

Full unedited gel for Figure 3J

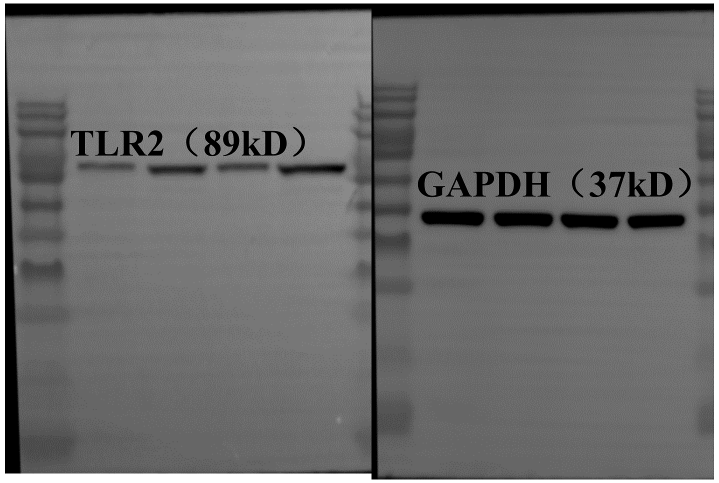

Full unedited gel for Figure 3K

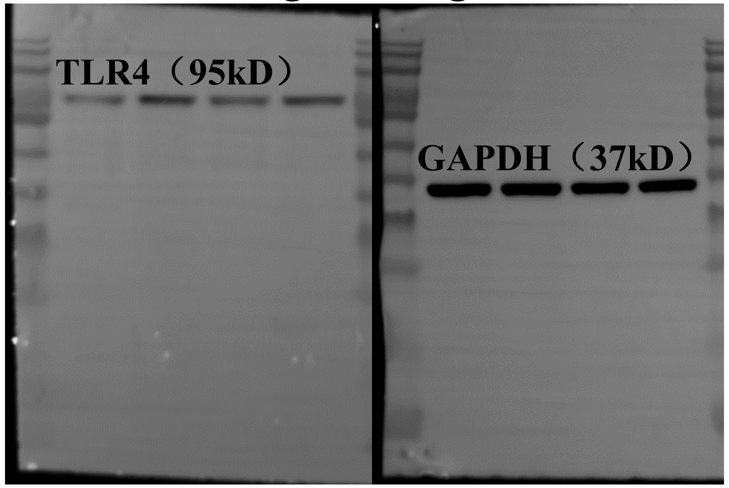

Full unedited gel for Figure 3L

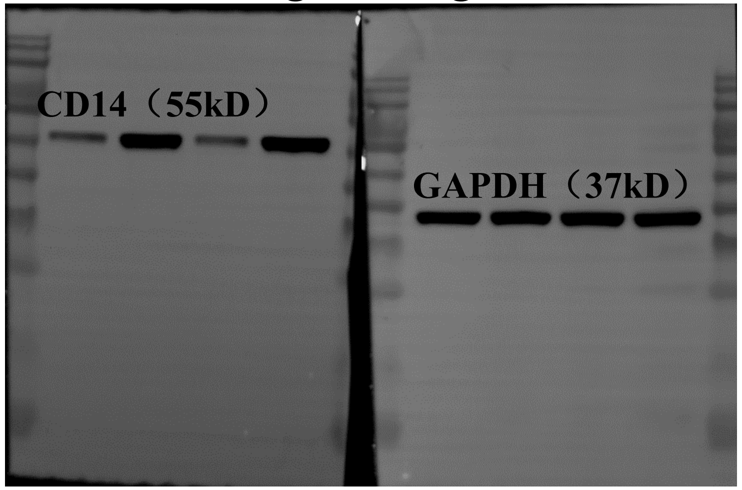

Full unedited gel for Figure 4A

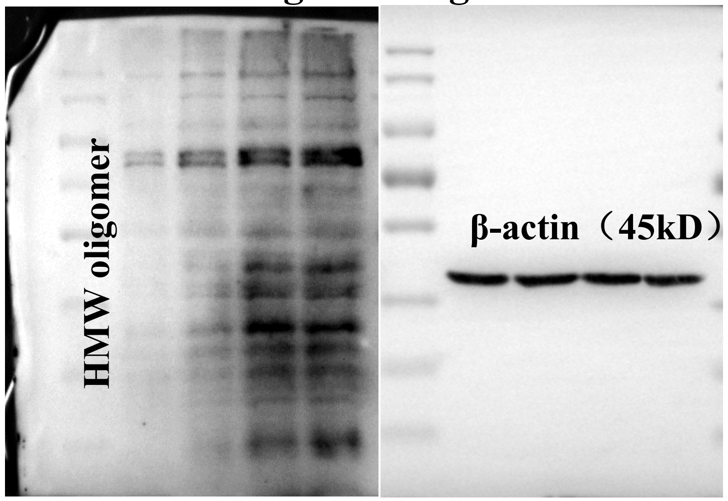

Full unedited gel for Figure 4B

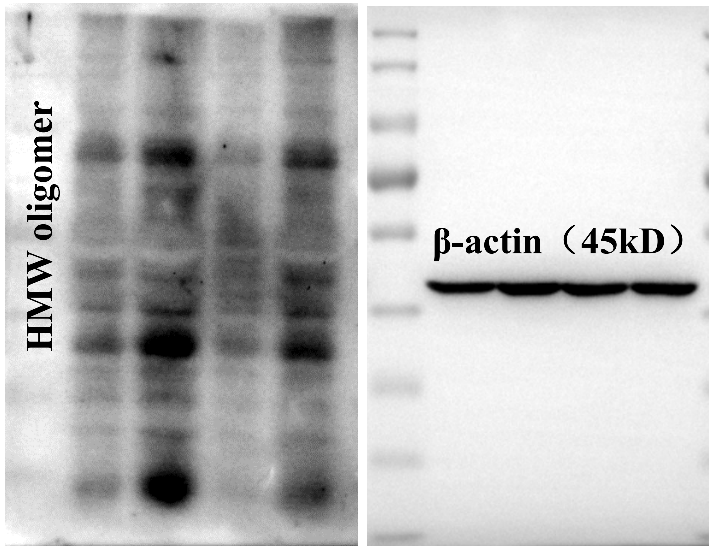

Full unedited gel for Figure 5A

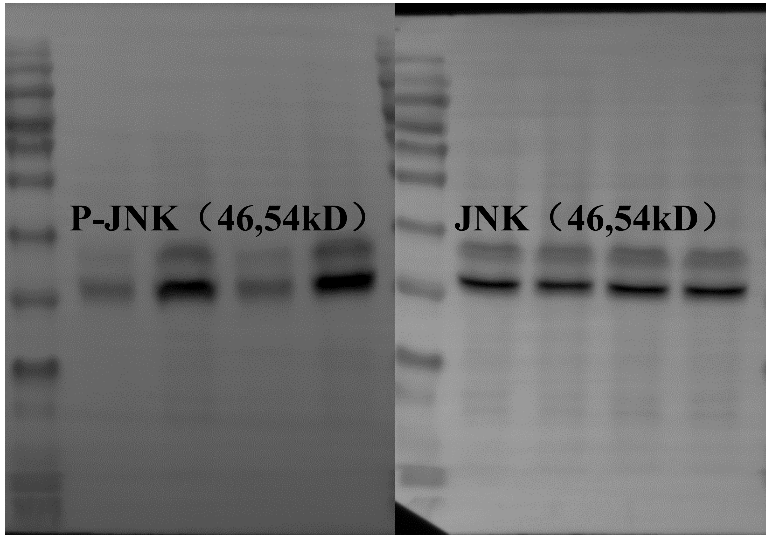

Full unedited gel for Figure 5B

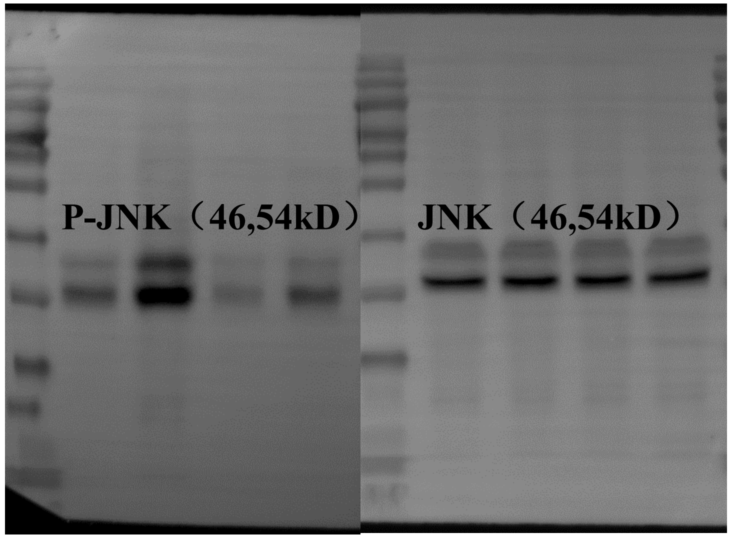

Full unedited gel for Figure 5C

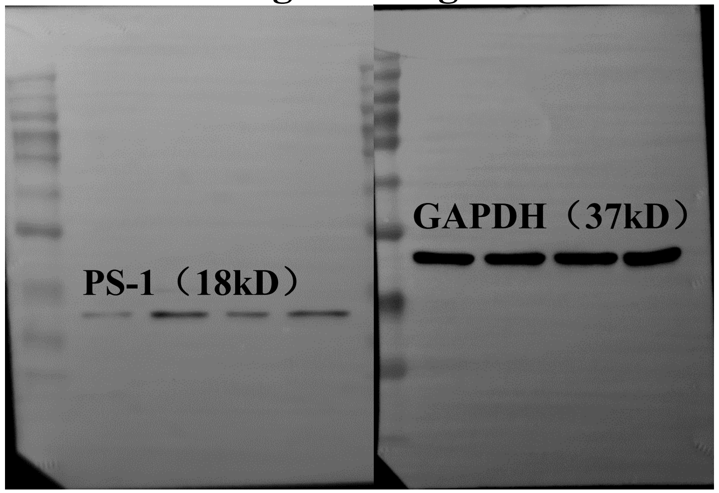

Full unedited gel for Figure 5D

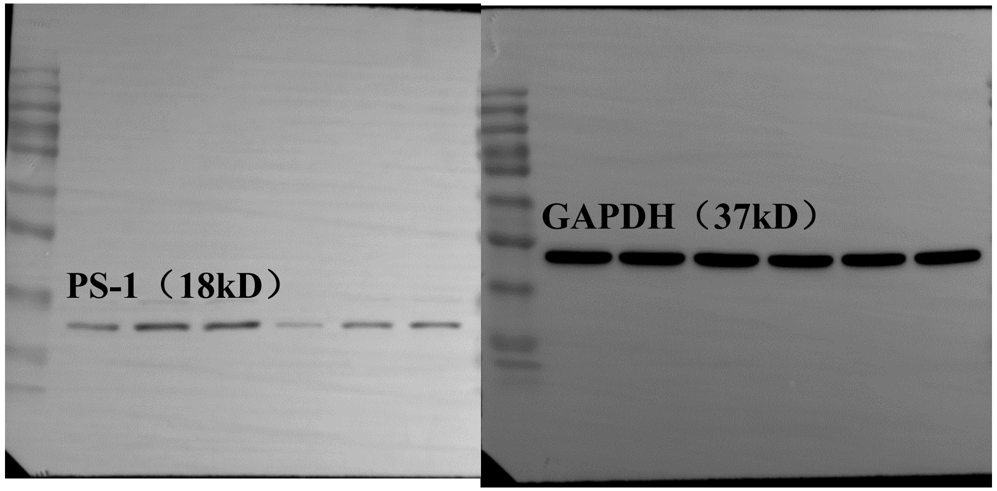

Full unedited gel for Figure 5E

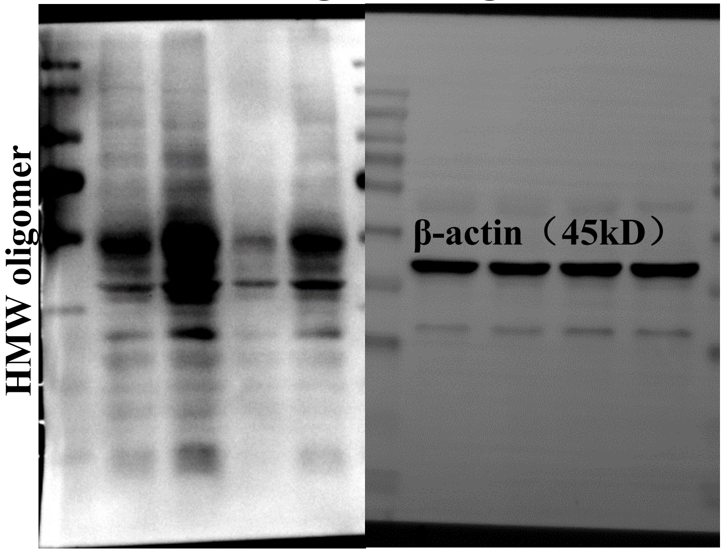

Full unedited gel for Figure 6A

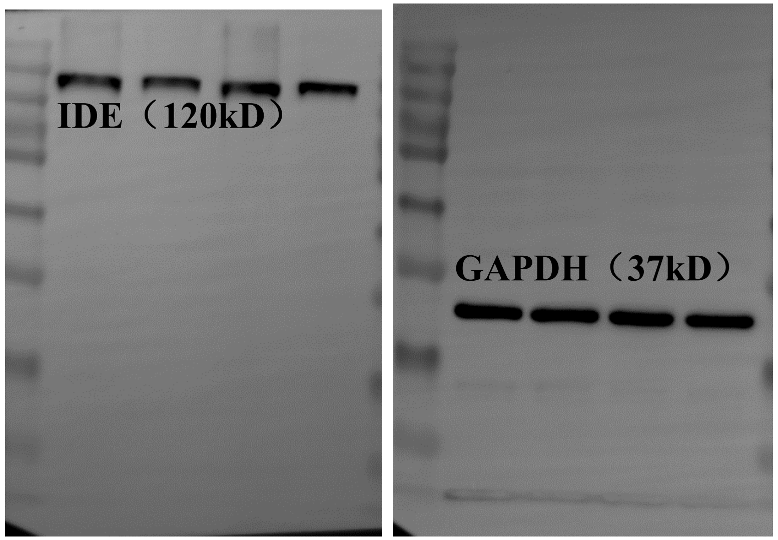

Full unedited gel for Figure 6B

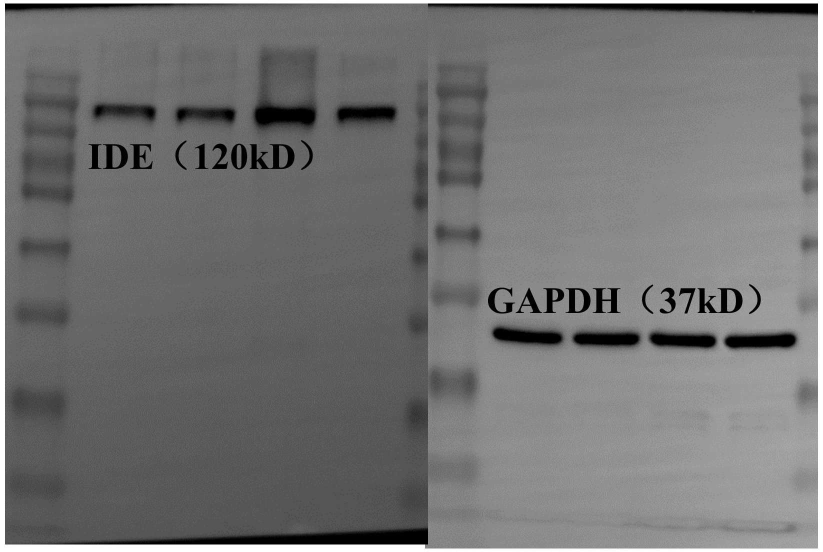

Full unedited gel for Figure 6C

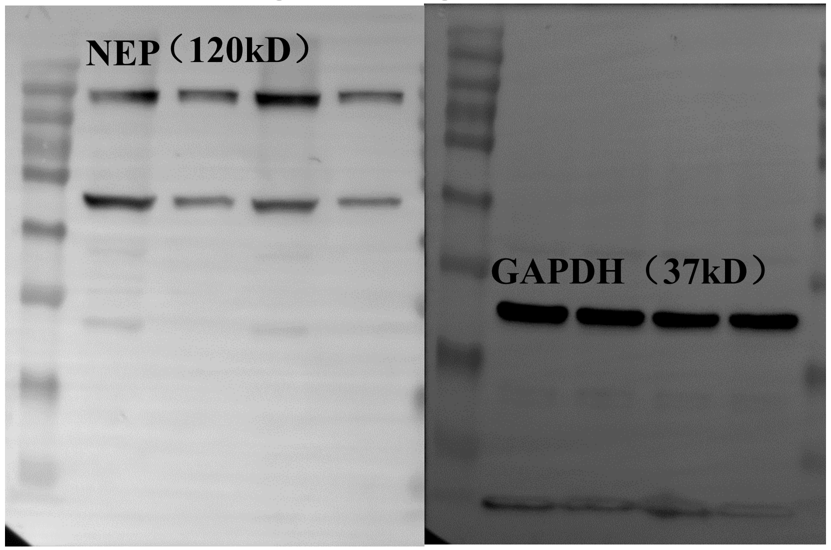

Full unedited gel for Figure 6D

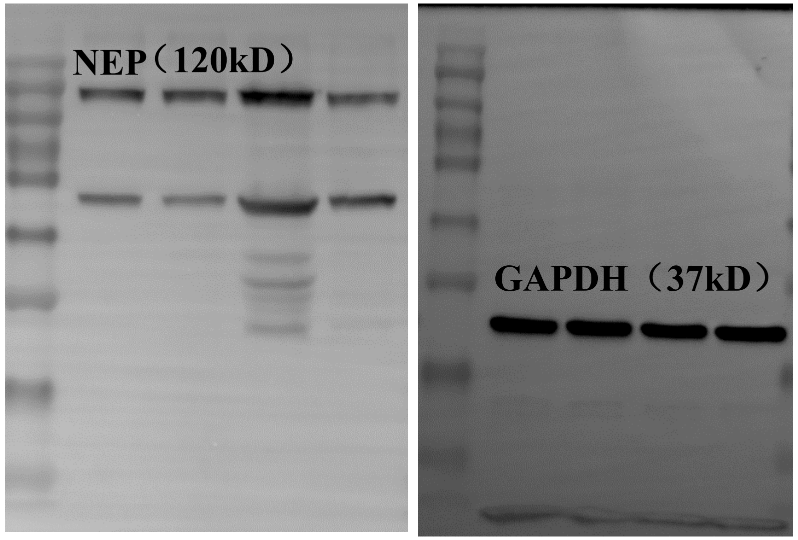

Full unedited gel for Figure 6E

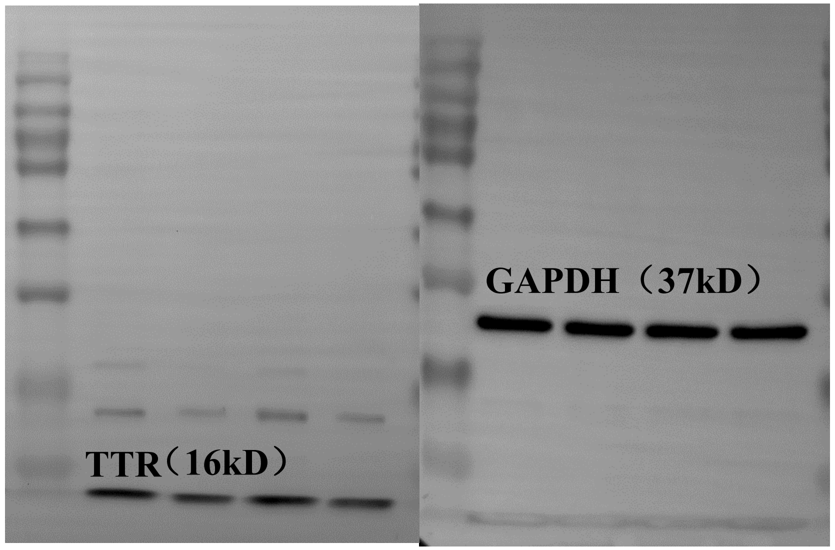

Full unedited gel for Figure 6F

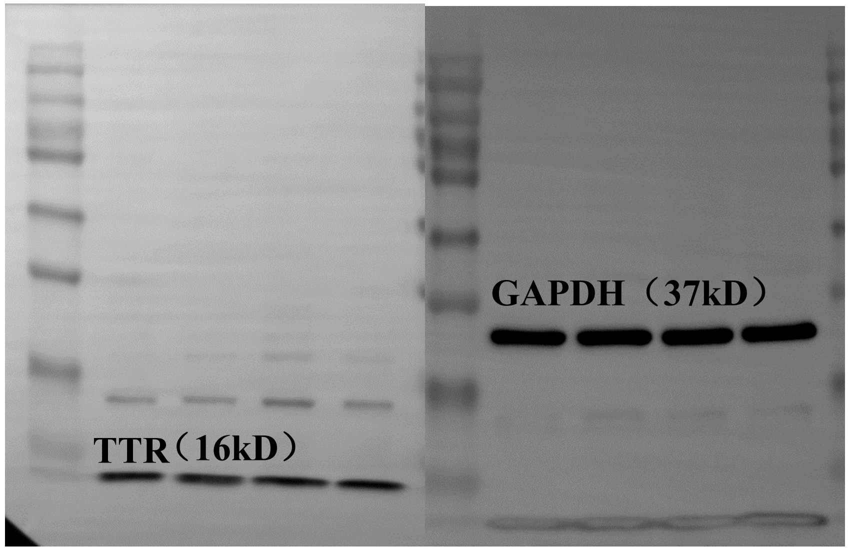

Supplement: Supplementary file 2 — Data S1. [file CNS-30-e70127-s001.zip › R1-WB unedited gels.pdf]
